# LINC00452 overexpression reverses oxLDL-induced injury of human umbilical vein endothelial cells (HUVECs) *via* regulating miR-194-5p/IGF1R axis

**DOI:** 10.3389/fcvm.2022.975640

**Published:** 2022-09-09

**Authors:** Liang Yuan, Dajie Wang, Zhaofeng Zhou

**Affiliations:** ^1^Department of Cardiology, First Affiliated Hospital of Nanjing Medical University, Nanjing, China; ^2^Department of Cardiology, Yancheng School of Clinical Medicine of Nanjing Medical University (Yancheng Third People's Hospital), Yancheng, China

**Keywords:** atherosclerosis, LINC00452, miR-194-5p, IGF1R, apoptosis

## Abstract

It has been reported that atherosclerosis (AS) is the basis of the development of coronary artery disease (CAD). In addition, a previous study demonstrated that long non-coding RNA LINC00452 was notably downregulated in the whole blood of patients with CAD. However, the role of LINC00452 in the progression of AS remains unclear. Therefore, to mimic AS *in vitro*, HUVECs were treated with 100 μg/ml oxLDL for 24 h. Reverse transcription-quantitative PCR was performed to detect the expression levels of LINC00452 and IGF1R in HUVECs. Additionally, the cell angiogenetic ability was assessed by tube formation assay, while dual-luciferase reporter assay was carried out to explore the association among LINC00452, miR-194-5p, and IGF1R. The results showed that LINC00452 was downregulated in oxLDL-treated HUVECs. In addition, HUVEC treatment with oxLDL significantly inhibited cell viability, proliferation, and angiogenesis. However, the above effects were all reversed by LINC00452 overexpression. Furthermore, LINC00452 overexpression in HUVECs remarkably inhibited oxLDL-induced cell apoptosis and endothelial to mesenchymal transition. In addition, LINC00452 overexpression could markedly reverse oxLDL-induced inhibition of angiogenesis in HUVEC. The results of dual-luciferase reporter assay indicated that LINC00452 could bind with miR-194-5p. In addition, IGF1R was identified as a downstream target of miR-194-5p. And LINC00452 was able to regulate the miR-194-5p/IGF1R axis in HUVECs. Moreover, LINC00452 overexpression obviously reversed oxLDL-mediated growth inhibition of HUVEC *via* regulating the miR-194-5p/IGF1R axis. Overall, the current study demonstrated that LINC00452 overexpression reversed oxLDL-induced growth inhibition of HUVECs *via* regulating the miR-194-5p/IGF1R axis, thus providing a potential beneficial targets for AS.

## Introduction

Coronary artery disease (CAD) is the leading cause of mortality in patients with cardiovascular diseases (1). Coronary atherosclerosis (AS), characterized by deposition of lipids, complex sugars and other substances on the walls of the arteries (2), is considered to be the most common cause of CAD (3). The development of AS includes several pathological processes, including vascular endothelial cell injury, and the migration of smooth muscle cells (4–6).

Oxidized low-density lipoprotein (oxLDL) exhibits pro-inflammatory properties that can cause intracellular lipid deposition and foam-like changes (7). It has been reported that oxLDL can cause human umbilical vein endothelial cell (HUVEC) injury and dysfunction (8). Currently, the most common treatment approaches for AS include diet self-regulation and drug therapy (9–12). However, the effectiveness of the above treatment strategies remains unsatisfactory. Therefore, more effective treatments are urgently needed.

Long non-coding RNAs (lncRNA), a type of non-coding RNAs (ncRNAs), with a transcript length of exceed 200 nt, which play significant roles in the progression of AS (13, 14). In addition, microRNAs (miRNAs), another type of ncRNAs, can directly regulate the expression of messenger RNA (mRNA) *via* affecting their stability (15). According to the competitive endogenous RNA (ceRNA) network, R lncRNAs could sponge miRNAs (16), which could indirectly regulate the target mRNAs of miRNAs (17). For example, lncRNA Kcnq1 knockdown could inhibit the progression of AS *via* upregulating microRNA (miR)-452-3p (18). In addition, lncRNA NEXN-AS1 overexpression notably suppressed the development of AS *via* targeting Nexilin F-actin binding protein (19). Another study revealed that lncRNA LINC00452 was significantly downregulated in the whole blood of patients with CAD compared with healthy controls (14). In addition, the level of LINC00452 was much lower in patients with myocardial infarction, compared with that in patients with CAD (14). Moreover, the progression of CAD could contribute to the occurrence of myocardial infarction (20). Thus, these backgrounds suggested that LINC00452 may be closely associated with the progression of AS (14). Nevertheless, the function of LINC00452 in AS remains unclear. Moreover, the relationship between LINC00452 and miRNA in AS is needed to be explored.

Insulin-like growth factor 1 receptor (IGF-1R) is known to be a key mediator in cell growth, which could positively regulate Akt signaling (21, 22). In addition, IGF-1R was able to participate in the progression of AS as its upregulation could reverse oxLDL-induced injury of HUVECs (23). Thus, IGF-1R could be served as a suppressor in AS. Nevertheless, the relation between LINC00452 and IGF-1R in AS remains unclear.

Based on the above backgrounds, this study aimed to investigate the function of LINC00542 in AS. We hope this research would provide a novel theoretical basis for exploring new strategies against AS.

## Materials and methods

### Cell culture and treatment

Human umbilical vein endothelial cells (HUVECs) were obtained from the American Type Culture Collection (ATCC; cat.: CRL-1730) and cultured in DMEM (iCell Bioscience Inc.) at 37°C and 5% CO_2_. OxLDL was purchased from Beijing Solarbio Science & Technology Co. Ltd. in a lyophilized powder form and it was reconstituted with deionized water at a store concentration of 1 mg/ml. HUVECs were treated with 100 μg/ml oxLDL for 24 h.

### Cell transfection

The pcDNA3.1 control vector (NC), the pcDNA3.1-LINC00452 overexpression plasmid (LINC00452 OE; accession number: BC034570.1), miR-194-5p agomir, miR-194-5p agomir control (agomir-ctrl) and small interfering RNA (siRNA) targeting LINC00452 and insulin-like growth factor 1 receptor (IGF1R) were synthesized by Shanghai GenePharma Co., Ltd. HUVECs were transfected with pcDNA3.1-NC (1 μg/μl), pcDNA3.1-LINC00452 (1 μg/μl), miR-194-5p agomir (50 nM), agomir-ctrl (50 nM), LINC00452 siRNA1 (10 nM), LINC00452 siRNA2 (10 nM), IGF1R siRNA (10 nM), or siRNA control (siRNA-ctrl; 10 nM) using Lipofectamine^®^ 2000 (Thermo Fisher Scientific, Inc.) according to the manufacturer's protocol. The targeted sequence of IGF1R siRNA was 5′-CAGTGATCACTTTGCAGCCTCCATG-3′ and that of siRNA-control was 5′-GATCATGCACAGTGCCTCTTCCATG-3′. In addition, the sequences of miR-194-5p agomir and miR-194-5p agomir-ctrl were listed as follows (24): miR-194-5p agomir, 5′-UGUAACAGCAACUCCAUGUGGA-3′; miR-194-5p agomir-ctrl, 5′-UUCUCCGAACGUGCUACGUTT-3′.

### Reverse transcription-quantitative polymerase chain reaction (RT-qPCR)

Total RNA was extracted from HUVECs using a TRIzol^®^ reagent (ELK Biotechnology, Co., Ltd.) according to the manufacturer's instructions. Subsequently, total RNA was reverse transcribed into complementary DNA (cDNA) using the EntiLink™ 1st Strand cDNA Synthesis Kit (ELK Biotechnology, Co., Ltd.). qPCR was performed using the EnTurbo™ SYBR Green PCR SuperMix kit (ELK Biotechnology, Co., Ltd.) on the StepOne™ Real-Time PCR system (Thermo Fisher Scientific, Inc.). The thermocycling conditions were used as follows: 3 min at 95°C, followed by 40 cycles of 10 s at 95°C, 30 s at 58°C, and 30 s at 72°C. The primer sequences used were as follows: For β-actin, forward, 5′-GTCCACCGCAAATGCTTCTA-3′, and reverse, 5′-TGCTGTCACCTTCACCGTTC-3′; for LINC00452, forward, 5′-GTCCACTGTGAAGCTCGACG-3′, and reverse, 5′-GAGCACCACTCTGTCCACTCAG-3′; and for IGF1R, forward, 5′-GCATCATCATAACCTGGCACC-3′, and reverse, 5′-GTAAACGGCGTACTGAGTCCAG-3′. The RT-qPCR results were calculated using the 2^−ΔΔCq^ method (25), and β-actin was used as the internal control for normalization.

### Cell counting Kit-8 (CCK8) assay

A CCK8 assay was carried out to evaluate the viability of HUVECs. The CCK8 kit was purchased from Beyotime Institute of Biotechnology. Briefly, HUVECs in logarithmic growth phase were digested with trypsin and were then resuspended at a density of 5 × 10^4^ cells/ml. Subsequently, HUVECs were seeded into 96-well plates at a density of 5 × 10^3^ cells/well and cultured overnight at 37°C and 5% CO_2_. The next day, HUVECs were treated with 100 μg/ml oxLDL and incubated for 24 h. The medium was then replaced and cells were cultured in complete medium for 24, 48, and 72 h. Subsequently, each well of the 96-well plate was supplemented with 10 μl CCK-8 solution followed by incubation at 37°C for 2 h. Finally, the absorbance at a wavelength of 450 nm was measured using a microplate reader (Thermo Fisher Scientific, Inc.).

### EdU (5-ethynyl-2'-deoxyuridine) staining

The proliferation ability of HUVECs was assessed using an EdU Detection kit (Guangzhou RiboBio Co., Ltd.). Briefly, HUVECs were incubated with 50 μM EdU for 2 h in an incubator at a constant temperature of 37°C. Subsequently, HUVECs were incubated with Apollo staining solution for 30 min at 37°C in the dark. The EdU positive cells were observed and counted in three randomly selected fields under a fluorescence microscope (Olympus Corporation).

### Cell apoptosis assays

The apoptosis rate of HUVECs was determined using an Annexin V-FITC Apoptosis Detection Kit (Beyotime Institute of Biotechnology). Briefly, HUVECs at a density of 5 × 10^4^ cells/well were seeded into 6-well plates and were then incubated with 5 μl Annexin V-FITC for 10 min and 5 μl PI for 5 min in the dark. Finally, HUVEC apoptosis was detected using a flow cytometer (Becton, Dickinson and Company). Early apoptosis (Annexin V-FITC positive cells) and late cell apoptosis (Annexin V-FITC/PI double positive cells) were counted.

### Transwell assay

To investigate the migration ability of HUVECs, a Transwell assay was performed. Briefly, HUVECs in fetal bovine serum (FBS)-free culture medium were supplemented into the upper chamber of the Transwell insert (pore size, 8 μm; Corning, Inc.), while the lower chamber was filled up with 600 μl complete medium supplemented with 10% FBS. Subsequently, HUVECs were incubated for 24 h at 37°C and 5% CO_2_. The lower chamber was then removed and the transwell plate was washed with PBS. Finally, HUVECs were fixed with paraformaldehyde for 20 min, followed by staining with 0.1% crystal violet for 10 min. Images of the migrated cells were captured under a microscope.

### Western blot analysis

Total proteins were extracted from HUVECs using a Radio Immunoprecipitation Assay (RIPA) lysis buffer (Beyotime Institute of Biotechnology) and the protein concentrations were quantified using a bicinchoninic acid (BCA) kit (Aspen Biotechnology Co., Ltd.). Subsequently, proteins (40 μg) were separated by 10% sodium dodecyl sulfate polyacrylamide gel electropheresis (SDS-PAGE) and were then transferred onto polyvinylidene fluoride (PVDF) membranes. Following blocking with 3% skimmed milk for 1 h at room temperature, the membranes were incubated with primary antibodies overnight at 4°C. The primary antibodies used were the following: Anti-Vascular endothelial cadherin (VE-cadherin; dilution, 1:1,000; cat. no. ab33168), anti-CD31 (dilution, 1:1,000; cat. no. ab222783), anti-α-smooth muscle actin (α-SMA; dilution, 1:1,000; cat. no. ab150301), anti-IGF1R (dilution, 1:1,000; cat. no. ab182408), anti-β-catenin (dilution, 1:1,000; cat. no. ab32572) and anti-β-actin (dilution, 1:1,000; cat. no. ab8226; all from Abcam). Subsequently, the membranes were incubated with HRP-conjugated goat anti-rabbit IgG secondary antibody (dilution, 1:5,000; cat. no. ab205718; Abcam) for 1 h at room temperature (25). Finally, the blots were visualized using an enhanced chemiluminescence (ECL) kit (Thermo Fisher Scientific, Inc.). β-actin served as the internal control.

### RNA pull-down

For the RNA pulldown assay, the Biotin RNA Labeling Mix (Roche, Basel, Switzerland) was used to transcribe and label probe-control or probe-LINC00452. An RNA structure buffer (Thermo, MA, USA) was used to induce secondary structure formation from the biotin-labeled RNAs. The biotinylated LINC00452 and negative control (bio-NC) were generated *via* GenePharma and coated to streptavidin-conjugated magnetic beads. Cells were lysed and then incubated with the magnetic beads for 6 h. The RNA on the beads was isolated and the enrichment level of miR-194-5p was detected by RT-PCR.

### Tube formation assay

Matrigel obtained by Corning, Inc. [cat. no. 354248; high concentration (HC)] was used to evaluate the tube formation ability of HUVECs. Briefly, a 50 μl Matrigel homogenate was drawn using a precooled suction nozzle and was then spread on the surface of the culture plate (24-well). HUVECs were treated with 100 μg/ml oxLDL or/and LINC00452 OE for 24 h. Subsequently, cells (1 × 10^5^) were seeded into the Matrigel-coated wells and incubated for 12 h at 37°C and 5% CO_2_. The formed tubes were observed and images were captured at three randomly selected fields under a microscope. Finally, the number of branch points and capillary length were measured using ImageJ software.

### Oil-red O staining

Lipid accumulation in HUVECs was detected using Oil-red O staining. Briefly, cell culture medium was carefully removed and HUVECs were fixed with 10% formaldehyde for 30 min. Subsequently, HUVECs were stained with Oil-red O staining for 10 min. In addition, cell nuclei were stained with 5 μg/μl Hoechst 33342 for 10 min. Finally, the oil-red-positive cells were observed and images were captured at three randomly selected fields under a microscope.

### Measurement of mitochondrial membrane potential (MMP)

A mitochondrial membrane potential assay kit with JC-1 (Beyotime Institute of Biotechnology) was used to evaluate MMP. Firstly, HUVECs were collected following cell treatment with 0.25% trypsin followed by washing twice with PBS. Subsequently, HUVECs were stained with 0.5 ml JC-1 staining solution for 20 min at 37°C and were then centrifugated at 200 g for 3 min at 4°C. Finally, the cells were washed twice with 1× JC-1 staining buffer, resuspended in 500 μl 1× JC-1 staining buffer and analyzed by flow cytometry.

### Dual-luciferase reporter assay

The partial sequences of LINC00452 and the 3'-untranslated region (3'-UTR) of IGF1R encompassing the putative binding sites for miR-194-5p were synthesized by Sangon Biotech Co., Ltd. These sequences were subcloned into the pmirGLO dual-luciferase miRNA target expression vector (Beyotime Institute of Biotechnology). Subsequently, the LINC00452/IGF1R (wild type, WT) or LINC00452/IGF1R (mutant, MUT) plasmids, together with the control, agomir-control (agomir-ctrl) or miR-194-5p agomir were transfected into HUVECs using Lipofectamine 2000^®^ for 48 h. The Dual Luciferase Reporter Assay System (Beyotime Institute of Biotechnology) was used to detect the luciferase activity of HUVECs.

### Statistical analysis

Data were presented as the mean ± standard deviation (*S.D*.). Differences between HUVECs and oxLDL-treated HUVECs were analyzed by un-paired Student's *t*-test. Differences between three or more groups were analyzed by One-way analysis of variance (ANOVA) and Tukey's tests. *P* < 0.05 was considered as statistically significant. Software Gradpad Prism was used to perform these analyses. All data were repeated independently at least three times.

## Results

### LINC00452 overexpression reverses oxLDL-induced growth inhibition of HUVECs

**Figure 1:**
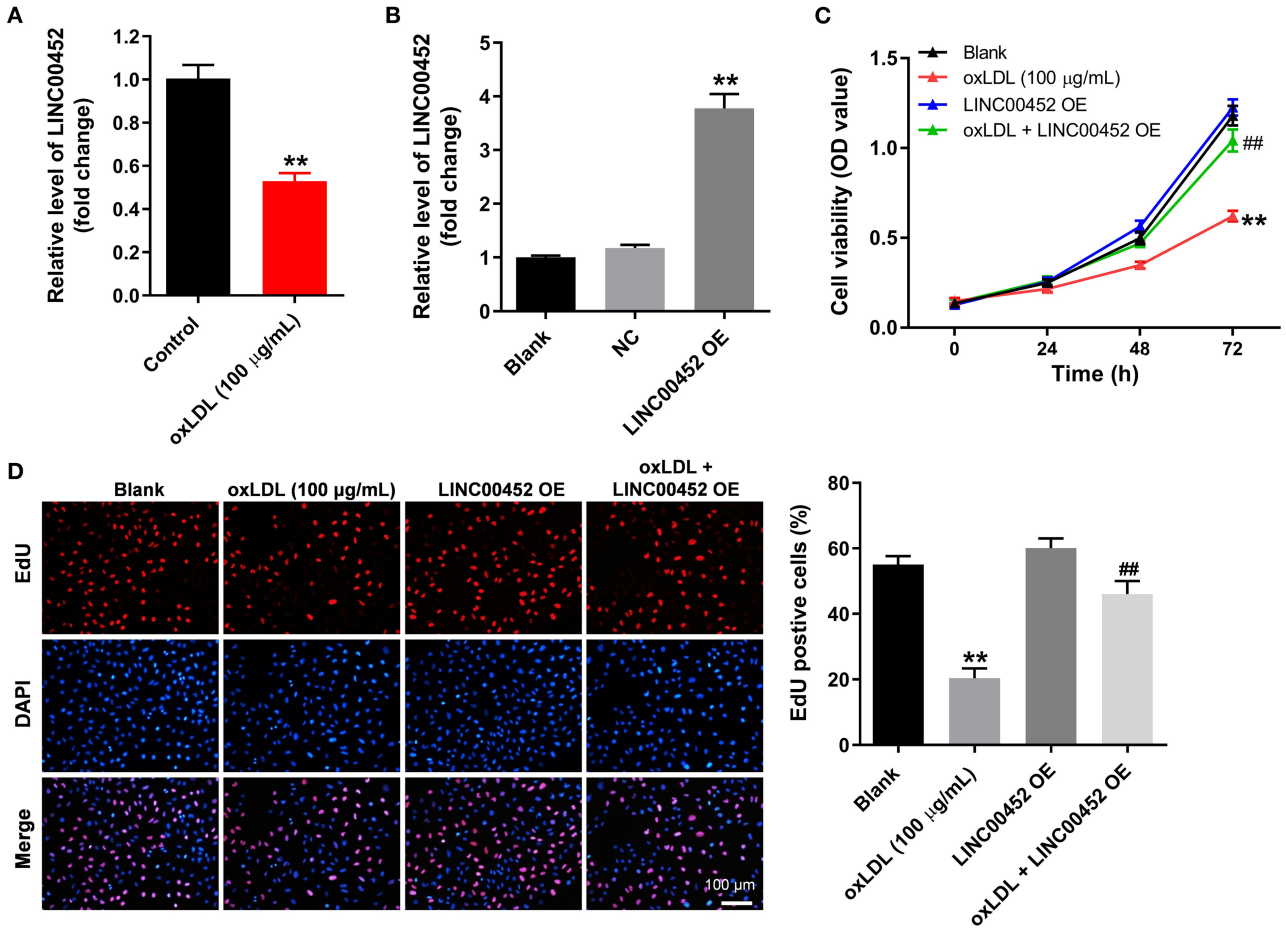
LINC00452 overexpression reverses oxLDL-induced growth inhibition of HUVECs. **(A)** HUVECs were treated with oxLDL (100 μg/mL) for 24 h. RT-qPCR was performed to detect the expression of LINC00452 in HUVECs. **(B)** HUVECs were transfected with pcDNA3.1-NC or pcDNA3.1-LINC00452 using Lipofectamine 2000 for 24 h. RT-qPCR was performed to detect the efficiency of transfection. **(C,D)** HUVECs were treated with oxLDL or/and pcDNA3.1-LINC00452. CCK8 and EdU assays were performed to evaluate the viability and proliferation of HUVECs, respectively. **P < 0.01 compared to the control group. ^##^*P* < 0.01 compared to the oxLDL (100 μg/mL) group, *n* = 3.

To mimic AS *in vitro*, HUVECs were treated with oxLDL for 24 h. As shown in Figure 1A, treatment with oxLDL significantly downregulated LINC00452 in HUVECs. HUVEC transfection with the pcDNA3.1-LINC00452 overexpression plasmid notably upregulated LINC00452 (Figure 1B), and the level of LINC00452 in HUVECs was notably inhibited by LINC00452 siRNAs (Supplementary Figure 1A). Since HUVECs were more sensitive to LINC00452 siRNA1, compared with LINC00452 siRNA2, LINC00452 siRNA1 was selected of use in subsequent analysis. In addition, oxLDL remarkably attenuated HUVEC viability, which was notably reversed following cell transfection with pcDNA3.1-LINC00452 (Figure 1C). In contrast, oxLDL-induced inhibition of HUVEC proliferation was further aggravated by LINC00452 silencing (Supplementary Figure 1B). Furthermore, oxLDL markedly inhibited the proliferation ability of HUVECs. However, the inhibitory effect of oxLDL was abolished by LINC00452 overexpression (Figure 1D). The above findings indicated that LINC00452 overexpression significantly reversed the oxLDL-induced growth inhibition of HUVECs.

### LINC00452 overexpression reverses oxLDL-induced apoptosis, migration and endothelial to mesenchymal transition (EndMT) of HUVECs

**Figure 2:**
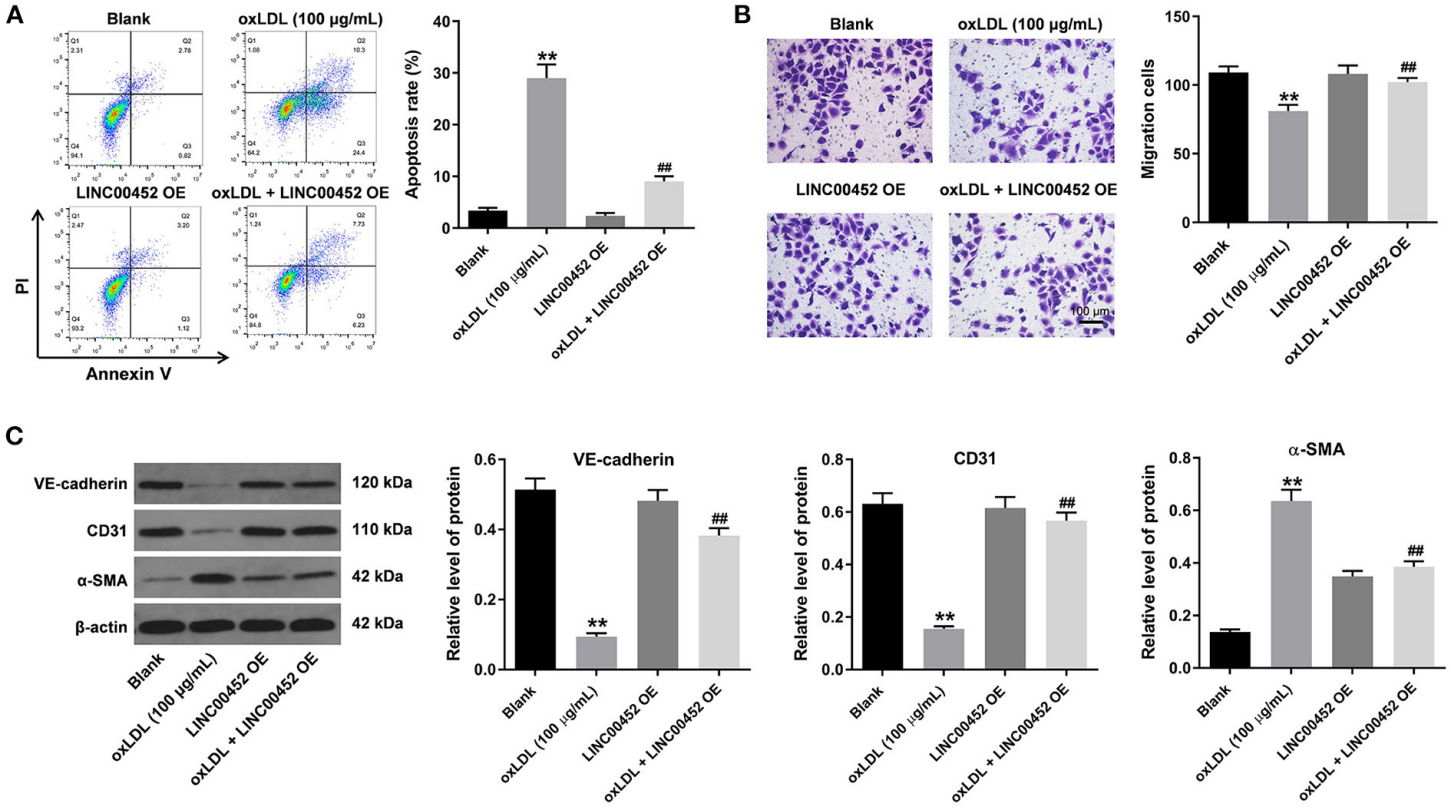
LINC00452 overexpression reverses oxLDL-induced apoptosis, migration and EndMT process of HUVECs. HUVECs were treated with oxLDL (100 μg/mL) for 24 h. Next, HUVECs were transfected with pcDNA3.1-LINC00452 using Lipofectamine 2000 for 24 h. **(A)** Flow cytometry assay was performed to detect the apoptosis of HUVECs. **(B)** Transwell assay was performed to detect the migration of HUVECs. **(C)** Western blot assay was performed to detect the expressions of α-SMA, CD31 and VE-cadherin in HUVECs. β-actin was used for normalization. **P < 0.01 compared to control group. ^##^*P* < 0.01 compared to oxLDL (100 μg/mL) group, *n* = 3.

To investigate the effect of LINC00452 overexpression on HUVEC apoptosis and migration, flow cytometric analysis and Transwell assays were performed. The results showed that treatment with oxLDL dramatically promoted HUVEC apoptosis, while this effect was notably abrogated by LINC00452 overexpression (Figure 2A). In addition, exposure to oxLDL notably decreased the migration ability of HUVECs. Consistently, the above effect was also reversed following cell transfection with the LINC00452 OE plasmid (Figure 2B). Subsequently, the protein expression levels of the EndMT-associated proteins, VE-cadherin, CD31 and α-SMA, were detected by western blot analysis (26, 27). The data revealed that HUVECs treatment with oxLDL obviously increased the protein expression levels of α-SMA and decreased those of VE-cadherin and CD31. However, the levels of the aforementioned proteins were notably restored following LINC00452 overexpression (Figure 2C). In summary, these data suggested that LINC00452 overexpression could remarkably reverse oxLDL-induced cell apoptosis, migration and EndMT in HUVECs.

### LINC00452 overexpression restores oxLDL-induced inhibition of HUVEC angiogenesis

**Figure 3:**
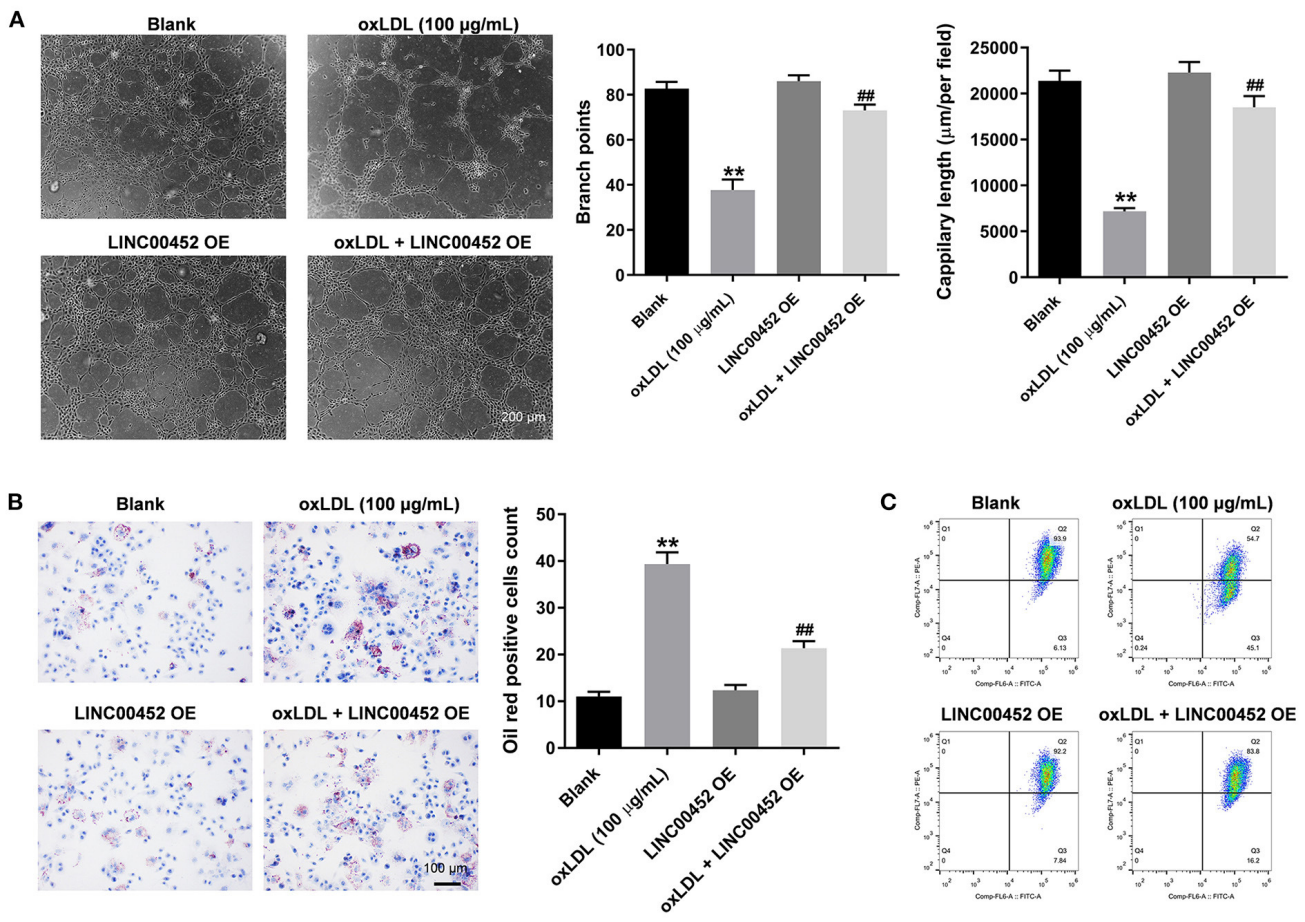
LINC00452 overexpression reverses oxLDL-induced inhibition of HUVECs angiogenesis. HUVECs were treated with oxLDL (100 μg/mL) for 24 h. Next, HUVECs were transfected with pcDNA3.1-LINC00452 using Lipofectamine 2000 for 24 h. **(A)** Tube formation assay was performed to measure the branch points and capillary length of HUVECs. **(B)** Oil-red O staining was used to detect the lipid accumulation in HUVECs. **(C)** Flow cytometry assay was performed to detect the level of MMP. **P < 0.01 compared to control group. ^##^*P* < 0.01 compared to the oxLDL (100 μg/mL) group, *n* = 3.

To investigate the effect of LINC00452 overexpression on the angiogenic ability of HUVECs, tube formation assay was carried out. As shown in Figure 3A, oxLDL remarkably inhibited the branch points and capillary length of HUVECs, which were restored after cell transfection with the pcDNA3.1-LINC00452 plasmid (Figure 3A). Furthermore, oil-red O staining demonstrated that exposure to oxLDL significantly increased lipid accumulation in HUVECs. LINC00452 overexpression also notably abolished the effect of oxLDL on lipid accumulation (Figure 3B). It has been reported that the changes in MMP are also associated with the promotion of cell apoptosis (28). Therefore, the degree of MMP in HUVECs was determined. The flow cytometry analysis results showed that the oxLDL-mediated decreased MMP was markedly restored by LINC00452 overexpression (Figure 3C). Taken together, the aforementioned findings indicated that LINC00452 overexpression could markedly reverse the oxLDL-triggered inhibition of HUVEC angiogenesis.

### LINC00452 regulates the miR-194-5p/IGF1R axis in HUVECs

**Figure 4:**
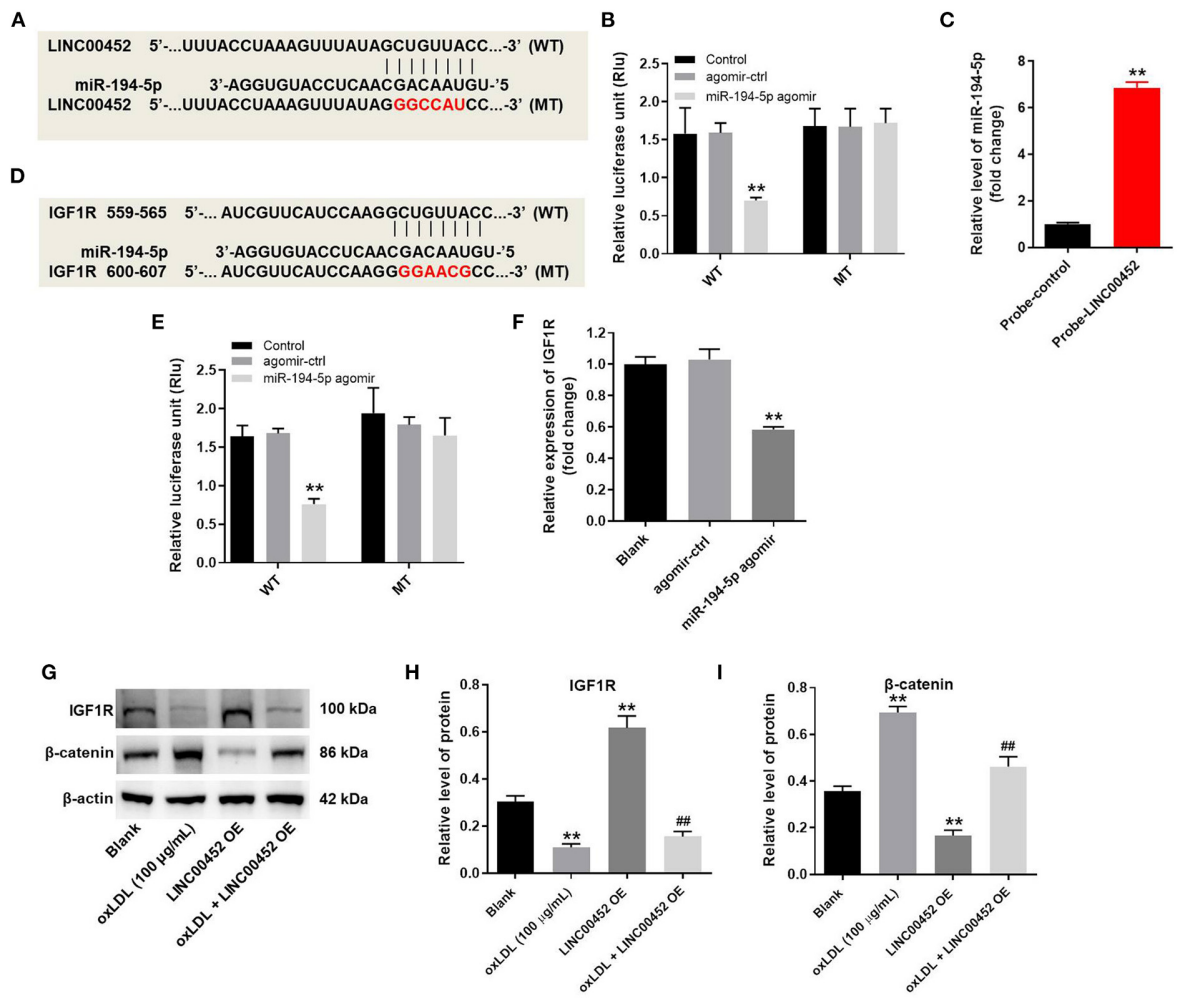
LINC00452 can regulate miR-194-5p/IGF1R axis in HUVECs. HUVECs were treated with oxLDL (100 μg/mL) for 24 h. Next, HUVECs were transfected with pcDNA3.1-LINC00452, miR-194-5p agomir NC, or miR-194-5p agomir using Lipofectamine 2000 for 24 h. **(A)** Starbase database was used to search the potential targets of LINC00452. **(B)** Dual-luciferase reporter assay was used to confirm the interaction between LINC00452 and miR-194-5p. **(C)** The enrichment of miR-194-5p in HUVECs was detected by RNA pull-down. **(D)** Targetscan database was used to predict the potential targets of miR-194-5p. **(E)** Dual-luciferase reporter assay was used to confirm the interaction between miR-194-5p and IGF1R. **(F)** RT-qPCR assay was performed to detect the level of IGF1R. **(G–I)** Western blot assay was performed to detect the expressions of IGF1R and β-catenin. β-actin was used for normalization. **P < 0.01 compared to control group. ^##^*P* < 0.01 compared to oxLDL (100 μg/mL) group, *n* = 3.

To explore the mechanism underlying the effect of LINC00452 on reversing oxLDL-mediated HUVEC growth inhibition, the potential targets of LINC00452 were predicted by bioinformatics analysis using the Starbase (http://starbase.sysu.edu.cn/) database. The analysis predicted that miR-194-5p could be a potential target of LINC00452 (Figure 4A). Emerging evidence has suggested that miR-194-5p is associated with the development of cardiovascular system-related diseases (29, 30). Therefore, miR-194-5p was further investigated. In addition, cell transfection with miR-194-5p agomir significantly reduced the luciferase activity of LINC00452 (WT), while it could not affect that of LINC00452 (MUT) (Figure 4B). The enrichment of miR-194-5p in HUVECs was significantly increased by probe-LINC00452 (Figure 4C). Subsequently, to identify the putative downstream targets of miR-194-5p, the TargetScan (http://www.targetscan.org/vert_71/) database was used. As shown in Figure 4D, IGF1R was predicted to exhibit a putative binding site with miR-194-5p. A previous study showed that IGF1R was closely associated with CAD (31). Therefore, IGF1R was selected for further analysis. As expected, miR-194-5p agomir markedly inhibited the activity of IGF1R (WT), but it had limited effect on IGF1R (MUT) (Figure 4E). Besides, miR-194-5p agomir markedly downregulated IGF1R (Figure 4F). Furthermore, cell treatment with oxLDL obviously downregulated IGF1R and upregulated β-catenin in HUVECs, while LINC00452 overexpression notably reversed the above effects (Figures 4G–I). In summary, the aforementioned findings indicated that LINC00452 could regulate the miR-194-5p/IGF1R axis in HUVECs.

### LINC00452 overexpression reverses oxLDL-induced HUVEC lesion *via* regulating the miR-194-5p/IGF1R axis

**Figure 5:**
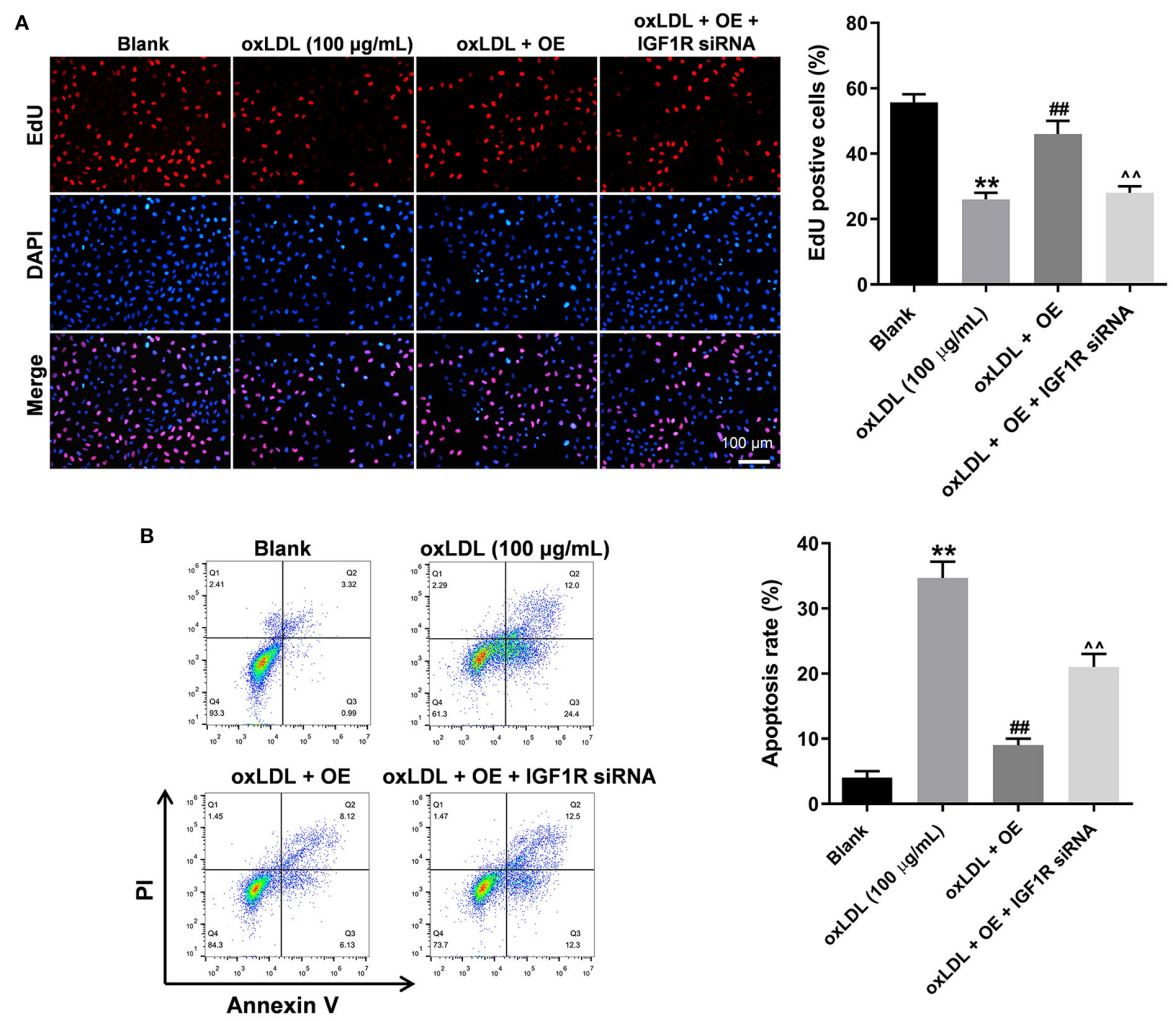
LINC00452 overexpression reverses oxLDL-induced HUVEC growth inhibition *via* regulating miR-194-5p/IGF1R pathway. HUVECs were treated with oxLDL (100 μg/mL) for 24 h. Next, HUVECs were transfected with pcDNA3.1-LINC00452 or/and IGF1R siRNA agomir using Lipofectamine 2000 for 24 h. **(A)** EdU assay was performed to detect the proliferation of HUVECs. **(B)** Flow cytometry assay was performed to detect the apoptosis of HUVECs. ^##^*P* < 0.01 compared to oxLDL (100 μg/mL) group. ^∧∧^*P* < 0.01 compared to oxLDL+LINC00452 OE group, *n* = 3.

**Figure 6:**
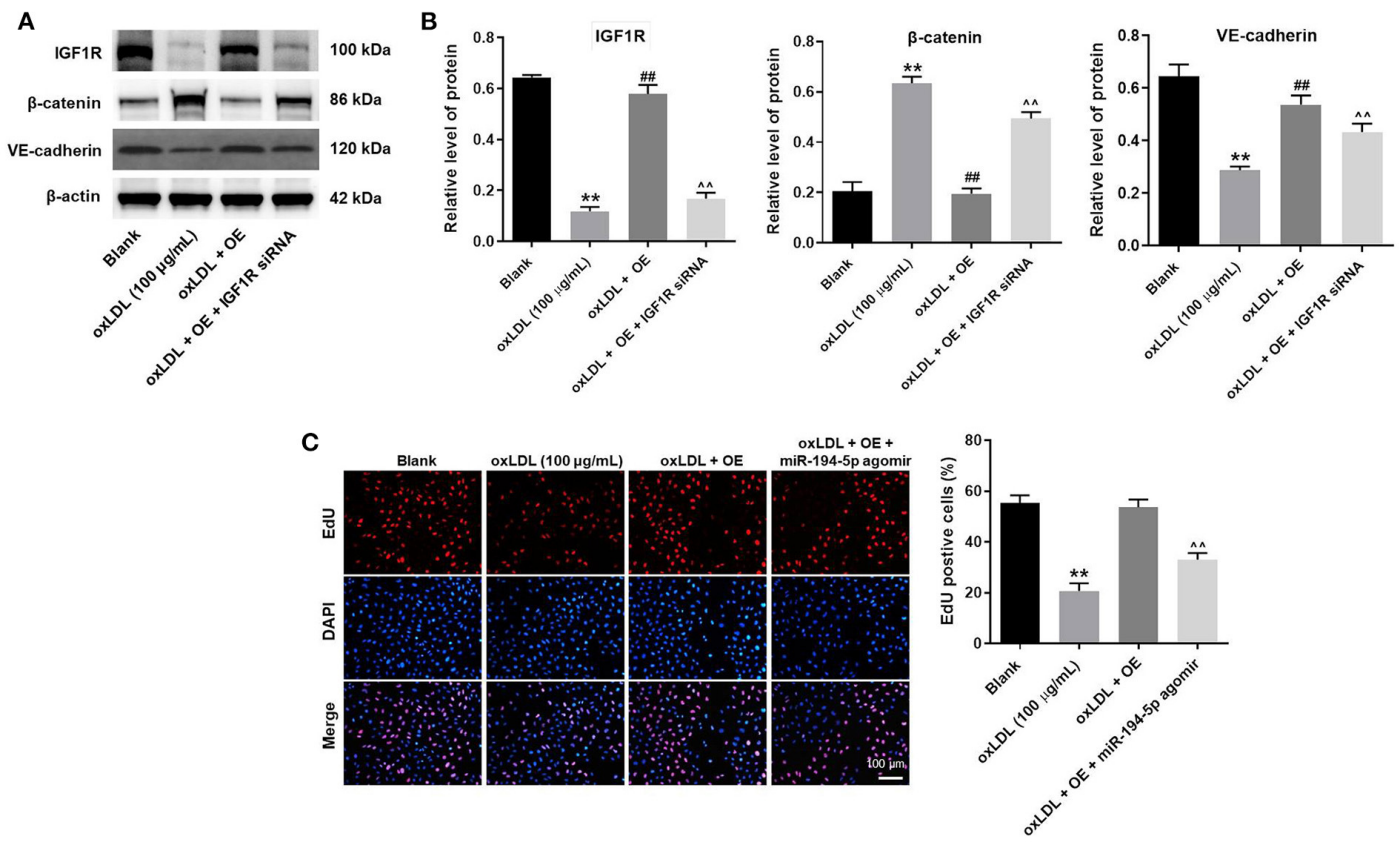
LINC00452 overexpression reverses oxLDL-induced HUVEC growth inhibition *via* regulating miR-194-5p/IGF1R pathway. **(A)** Western blot assay was performed to detect the expressions of IGF1R, β-catenin and VE-cadherin. **(B)** β-actin was used for normalization. **(C)** EdU assay was performed to detect the proliferation of HUVECs. **P < 0.01 compared to control group. ^##^*P* < 0.01 compared to oxLDL (100 μg/mL) group. ^∧∧^*P* < 0.01 compared to oxLDL+LINC00452 OE group, *n* = 3.

To further verify the mechanism underlying the effect of LINC00452 on regulating the growth of HUVECs, rescue experiments were performed. As shown in Figures 5A,B, LINC00452 overexpression restored oxLDL-induced growth inhibition of HUVECs, while the effect of LINC00452 overexpression was notably abolished by IGF1R silencing (Figure 5A). Consistently, the protective effect of LINC00452 overexpression against oxLDL was diminished following cell transfection with IGF1R siRNA. In addition, exposure to oxLDL significantly decreased the expression levels of IGF1R and VE-cadherin, and increased those of β-catenin in HUVECs. The expression levels of the above proteins were notably restored by LINC00452 OE (Figures 6A,B). The effect of LINC00452 overexpression on the expression levels of IGF1R, β-catenin and VE-cadherin were abolished by IGF1R knockdown (Figures 6A,B). Consistently, LINC00452 overexpression restored oxLDL-induced growth inhibition of HUVECs, while the effect of LINC00452 overexpression was notably abolished by miR-194-5p agomir (Figure 6C). Taken together, LINC00452 overexpression obviously reversed the oxLDL-mediated HUVEC growth inhibition *via* regulating the miR-194-5p/IGF1R axis.

## Discussion

It has been reported that LINC00452 is obviously downregulated in the whole blood of patients with CAD (14). Herein, LINC00452 was also downregulated in oxLDL-treated HUVECs. This finding was consistent with the aforementioned study, suggesting that LINC00452 could be involved in the development of AS.

Accumulating evidence has suggested that lncRNAs play a significant role in the pathology and development of AS (32). For example, lncRNA ANRIL was upregulated in patients with AS (32). In addition, lncRNA NORAD knockdown could also promote the development of AS (33). Furthermore, another study showed that lncRNA ZFAS1 overexpression inhibited cholesterol efflux and promoted inflammatory response in AS (34). Herein, LINC00452 overexpression could reverse oxLDL-induced growth inhibition of HUVECs.

It has been well-established that miR-194-5p is associated with the development of cardiovascular system-related diseases (29, 30). For example, a study in cardiac myocytes revealed that doxycycline attenuated cell autophagy *via* inhibiting the lncRNA H19/miR-194-5p/SIRT1 axis (35). Furthermore, a previous study demonstrated that IGF1R was also closely associated with cardiovascular system-related diseases (31). Additionally, it has been well-documented that IGF1R plays a significant role in the progression of myocardial infraction (31). In the current research, bioinformatics analysis using the Starbase and TargetScan databases predicted that LINC00452 could regulate the miR-194-5p/IGF1R axis. Furthermore, rescue experiments showed that LINC00452 overexpression notably reversed oxLDL-induced HUVEC apoptosis *via* regulating the miR-194-5p/IGF1R pathway. Consistently, the results of the present study indicated that miR-194-5p and IGF1R were involved in different pathways to regulate cardiovascular diseases. Thereby, a ceRNA network involving LINC00452/miR-194-5p/IGF1R was found in AS. LINC00452 could regulate the miR-194-5p, and miR-194-5p affect the expression of IGF1R. Overall, these findings could provide a better understanding of the role of LINC00452 in the progression of AS.

On the other hand, some other targets of miRNA-194-5p have been found. Sun et al. found mesenchymal stem cell extracellular vesicles-derived miR-194-5p could delay the development of intervertebral disc degeneration by targeting TRAF6 (36); Jia et al. suggested that miR-194-5p could promote TGFβ1-induced EMT, migration and invasion of tongue squamous cell carcinoma cells (37). Meanwhile, Zhang et al. found miR-194-5p could inhibit the cell growth through targeting JAK/STAT signaling (38), and our study was similar to this previous report. JAK/STAT signaling could contribute to the inflammatory responses, which was able to participate in the cell growth (39). Thus, the similar function between IGF-1R and JAK/STAT axis might contribute to the similarity between our research and Zhang et al.

Wnt/β-catenin pathway is a highly conserved signaling pathway in species evolution (40, 41). Emerging evidence has suggested that the Wnt/β-catenin pathway is widely involved in the development of AS (42, 43). For instance, Gong et al. (42) showed that STAT6 upregulation suppressed AS *via* activating the Wnt/β-catenin pathway. Additionally, Döring et al. (43) demonstrated that the C-X-C motif chemokine (CXC) ligand 12/CXC receptor 4 chemokine ligand/receptor axis inhibited the development of AS *via* also activating the Wnt/β-catenin axis. Besides, the proliferation of vascular smooth muscle cells in AS could be controlled *via* the Wnt/β-catenin axis (44). Herein, exposure of HUVECs to oxLDL significantly increased the expression levels of β-catenin, which were notably restored by LINC00452 OE. The current findings were consistent with previous studies suggesting that β-catenin could be associated with the progression of AS. Furthermore, it has been reported that IGF1R regulates β-catenin and is closely associated with the Wnt/β-catenin axis (45). In the current study, the expression levels of IGF1R were reduced, while those of β-catenin were enhanced in HUVECs exposed to oxLDL. The aforementioned findings implied that IGF1R could negatively regulate the β-catenin pathway in AS. That means, ceRNA network of LINC00452/miR-194-5p mediated the progression of AS might *via* β-catenin pathway.

Frankly speaking, the present study has some limitations. Firstly, only the LINC00452/miR-194-5p/IGF1R axis was investigated in the current study. However, other signaling pathways could be also regulated by LINC00452 in AS. Secondly, the effect of LINC00452 on vascular smooth muscle cells remains unclear. Therefore, further studies are urgently needed to evaluate the effect of LINC00452 on the above cells.

The strength (novelty) of this study was listed as follows: (1) the function of LINC00452 in ocLDL-treated HUVECs was firstly explored; (2) the relation between LINC00452 and miR-194-5p/IGF-1R axis in AS was firstly verified.

In summary, the strength of this study is that LINC00452 overexpression could reverse the oxLDL-induced growth inhibition of HUVECs *via* regulating the miR-194-5p/IGF1R axis. The above finding could provide a novel potential beneficial targets for AS.

## Data availability statement

The raw data supporting the conclusions of this article will be made available by the authors, without undue reservation.

## Author contributions

LY made major contributions to the conception, design, and manuscript drafting of this study. LY, DW, and ZZ was responsible for data acquisition, data analysis, data interpretation. DW and ZZ made substantial contributions to conception and design of the study and revised the manuscript critically for important intellectual content. All authors agreed to be accountable for all aspects of the work and approved the final manuscript.

## Conflict of interest

The authors declare that the research was conducted in the absence of any commercial or financial relationships that could be construed as a potential conflict of interest.

## Publisher's note

All claims expressed in this article are solely those of the authors and do not necessarily represent those of their affiliated organizations, or those of the publisher, the editors and the reviewers. Any product that may be evaluated in this article, or claim that may be made by its manufacturer, is not guaranteed or endorsed by the publisher.

## Supplementary material

Supplementary Figure 1 LINC00452 knockdown further aggravated oxLDL-induced inhibition of HUVEC proliferation. **(A)** HUVECs were transfected with LINC00452 siRNA1 or siRNA2. The level of LINC00452 in HUVECs was detected by RT-qPCR. **(B)** HUVECs were treated with oxLDL, LINC00452 siRNA1 or oxLDL + LINC00452 siRNA2. The proliferation of HUVECs was tested by EdU staining. ^**^*P* < 0.01 compared to control group. ^#^*P* < 0.05 compared to oxLDL (100 μg/mL) group.
