# Supplementary figures and images for: LINC00452 overexpression reverses oxLDL-induced injury of human umbilical vein endothelial cells (HUVECs) via regulating miR-194-5p/IGF1R axis
Source: Front Cardiovasc Med. 2022 Sep 9;9:975640. doi: 10.3389/fcvm.2022.975640 (PMC9500390; doi:10.3389/fcvm.2022.975640)

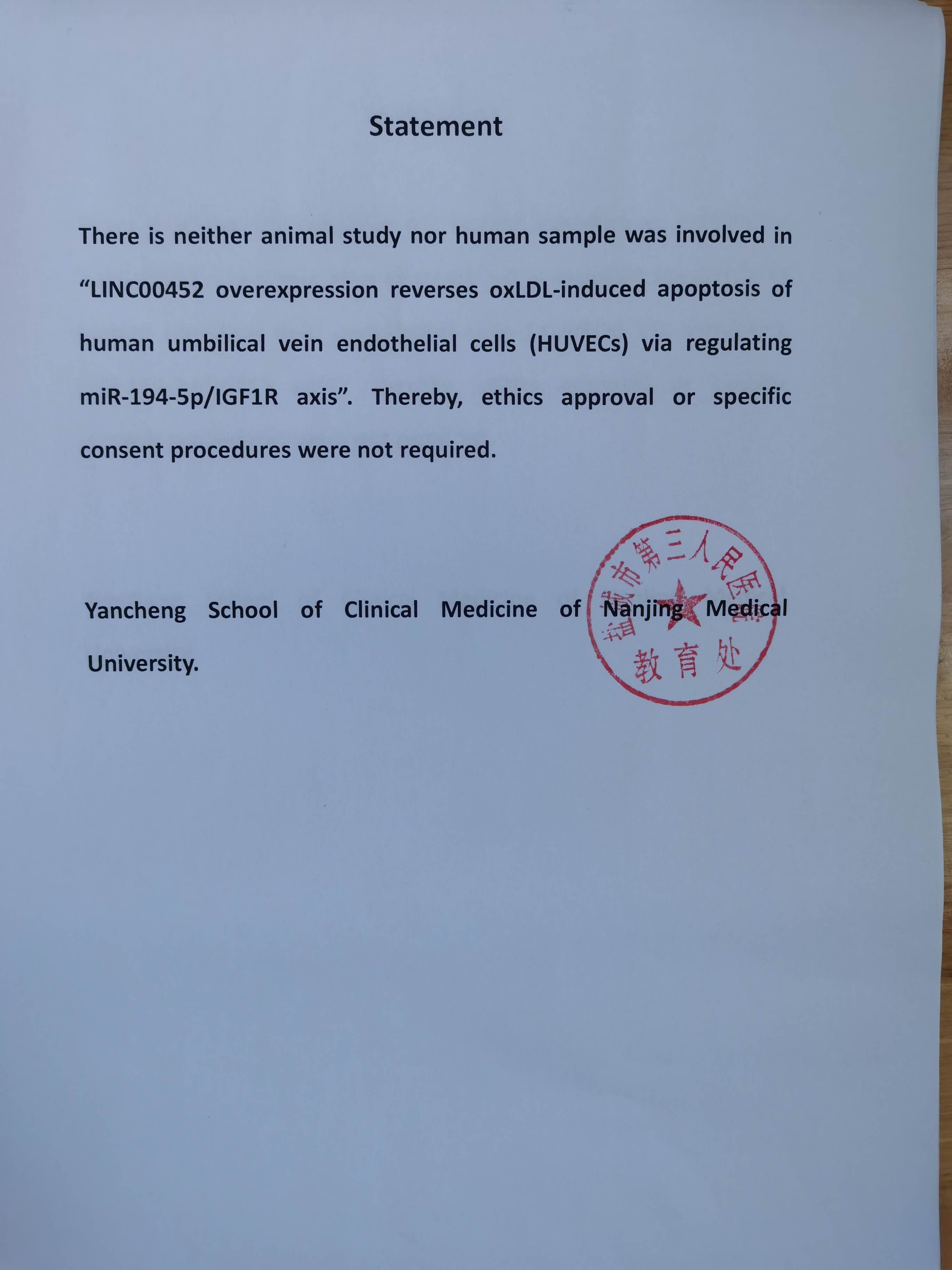

Supplement: Supplementary file 1 [file Data_Sheet_1.ZIP › 975640_SupMaterial/975640_SupMaterial/Editorial 1.JPEG]

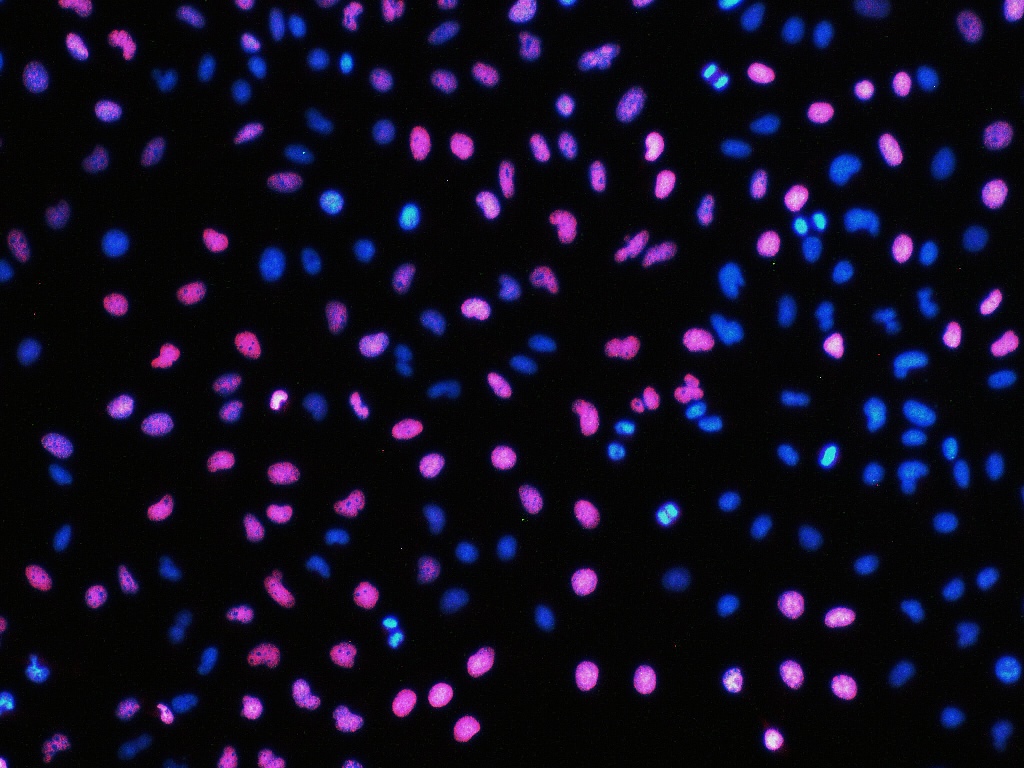

Supplement: Supplementary file 1 [file Data_Sheet_1.ZIP › 975640_SupMaterial/975640_SupMaterial/raw data frontiers/Figure 1/D/Blank 200-1+2.JPG]

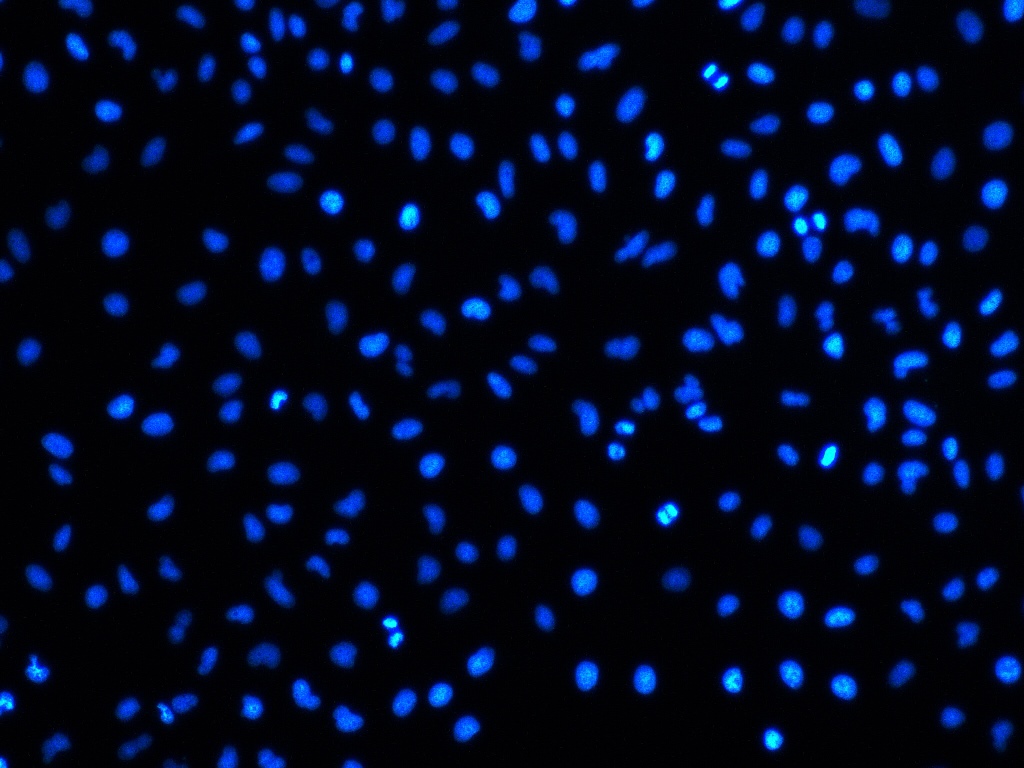

Supplement: Supplementary file 1 [file Data_Sheet_1.ZIP › 975640_SupMaterial/975640_SupMaterial/raw data frontiers/Figure 1/D/Blank 200-1.jpg]

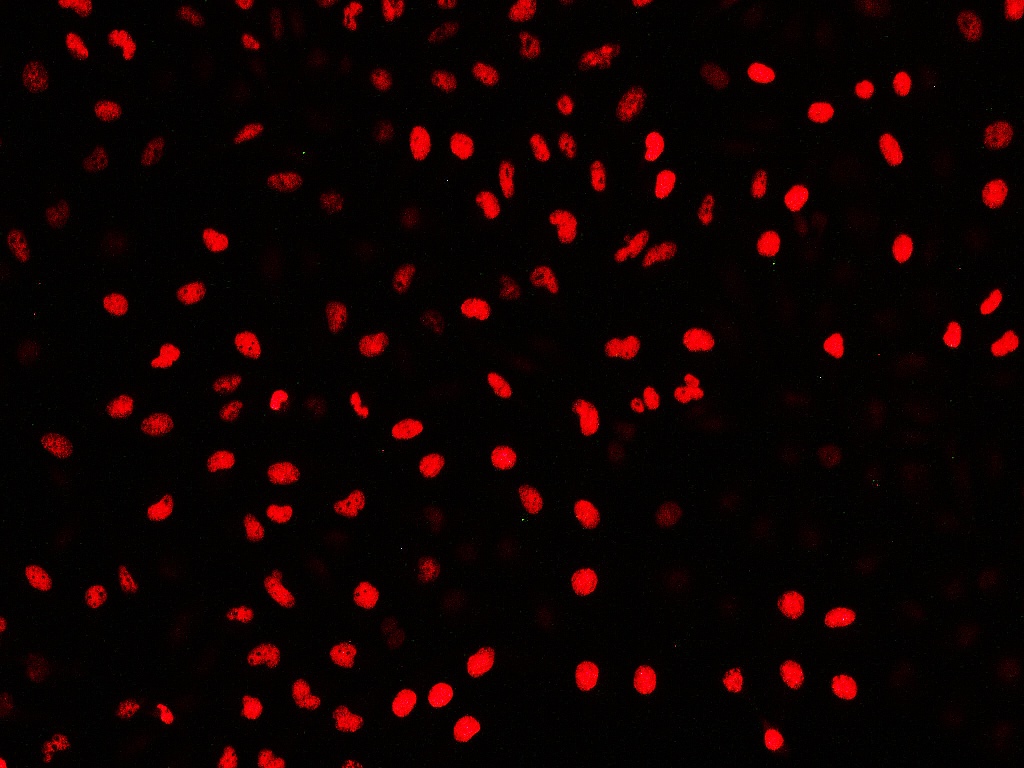

Supplement: Supplementary file 1 [file Data_Sheet_1.ZIP › 975640_SupMaterial/975640_SupMaterial/raw data frontiers/Figure 1/D/Blank 200-2.jpg]

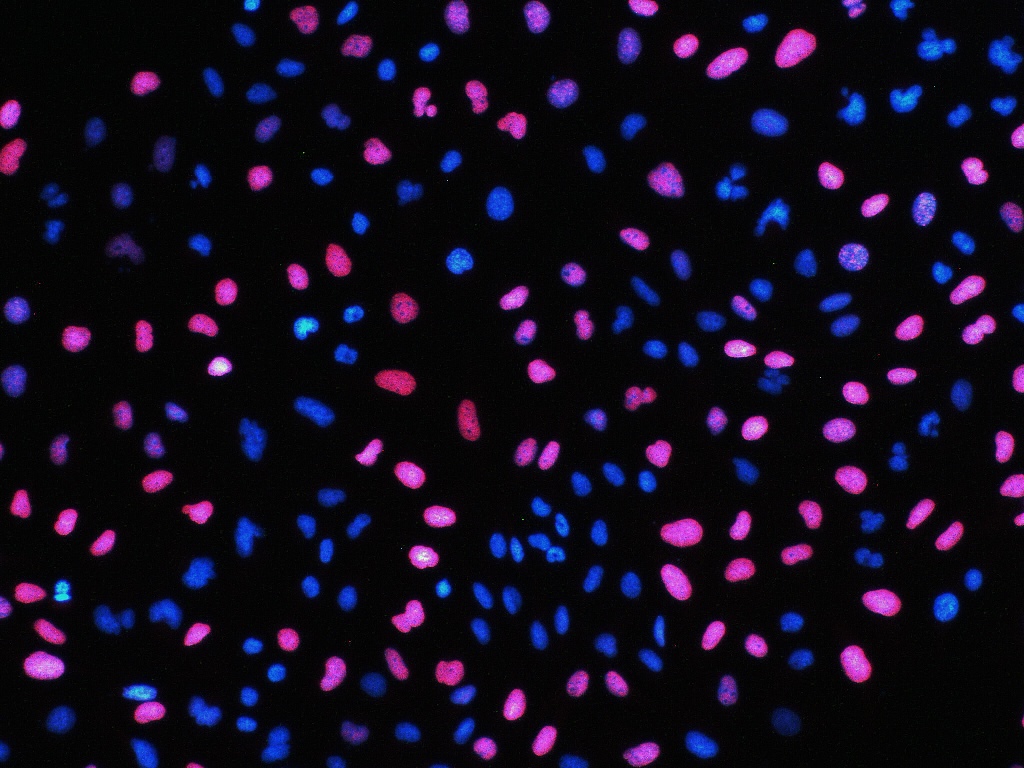

Supplement: Supplementary file 1 [file Data_Sheet_1.ZIP › 975640_SupMaterial/975640_SupMaterial/raw data frontiers/Figure 1/D/Linc00452-OE 200-1+2.JPG]

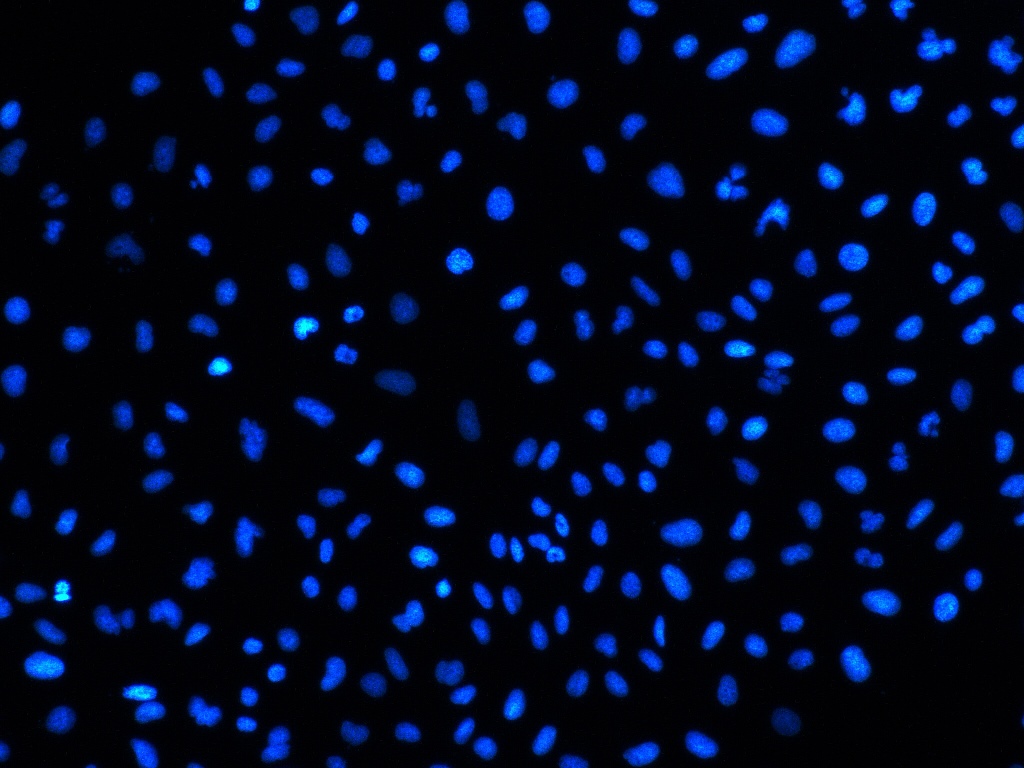

Supplement: Supplementary file 1 [file Data_Sheet_1.ZIP › 975640_SupMaterial/975640_SupMaterial/raw data frontiers/Figure 1/D/Linc00452-OE 200-1.jpg]

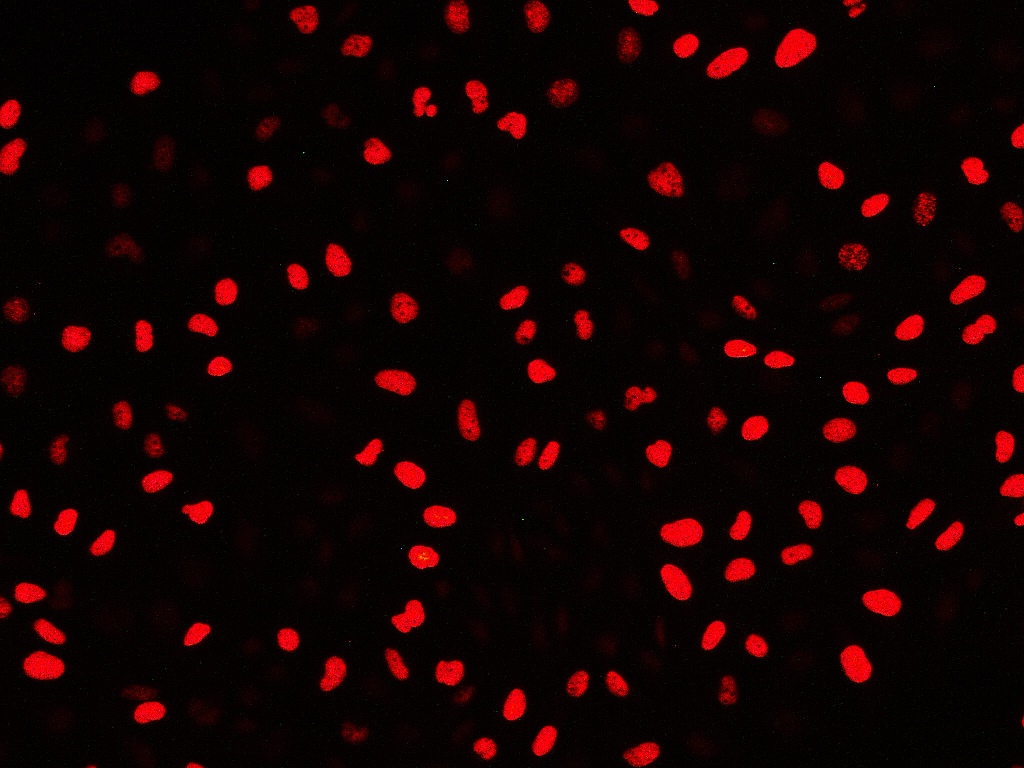

Supplement: Supplementary file 1 [file Data_Sheet_1.ZIP › 975640_SupMaterial/975640_SupMaterial/raw data frontiers/Figure 1/D/Linc00452-OE 200-2.jpg]

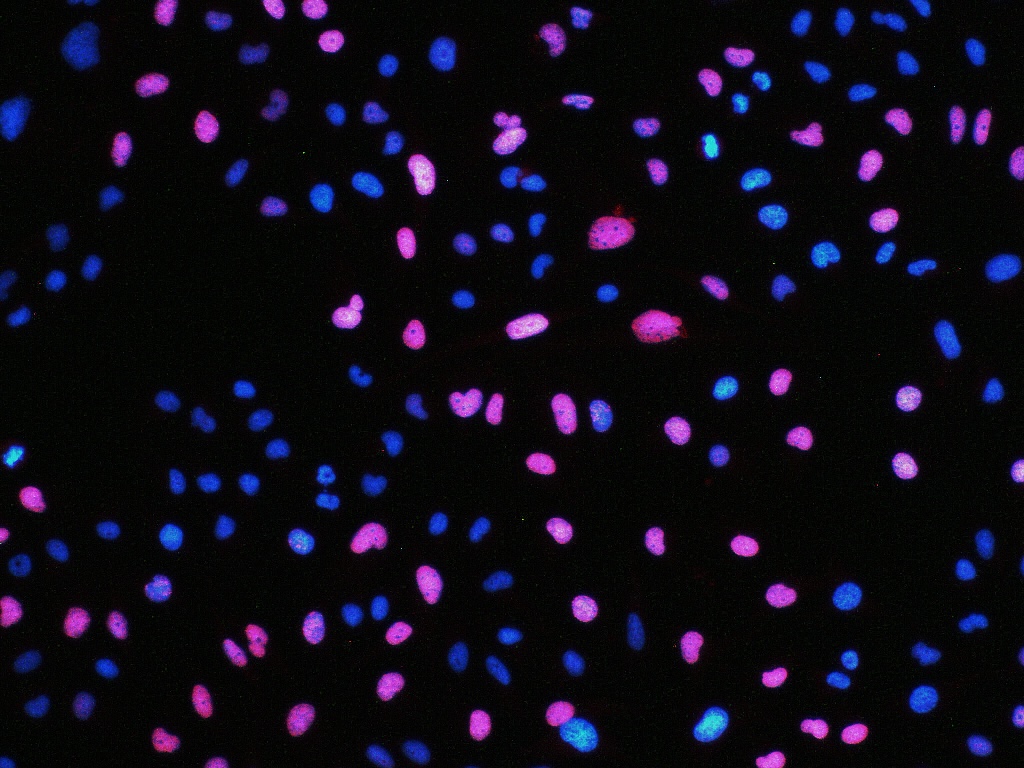

Supplement: Supplementary file 1 [file Data_Sheet_1.ZIP › 975640_SupMaterial/975640_SupMaterial/raw data frontiers/Figure 1/D/Linc00452-OE+Ox-LDL 200-1+2.JPG]

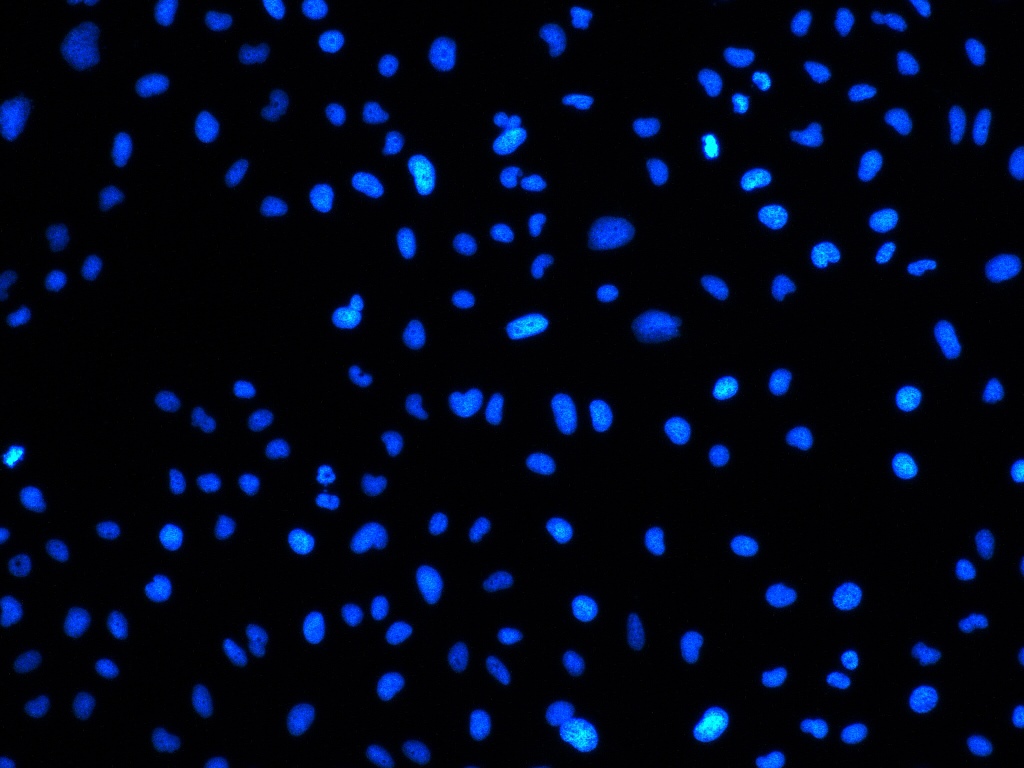

Supplement: Supplementary file 1 [file Data_Sheet_1.ZIP › 975640_SupMaterial/975640_SupMaterial/raw data frontiers/Figure 1/D/Linc00452-OE+Ox-LDL 200-1.jpg]

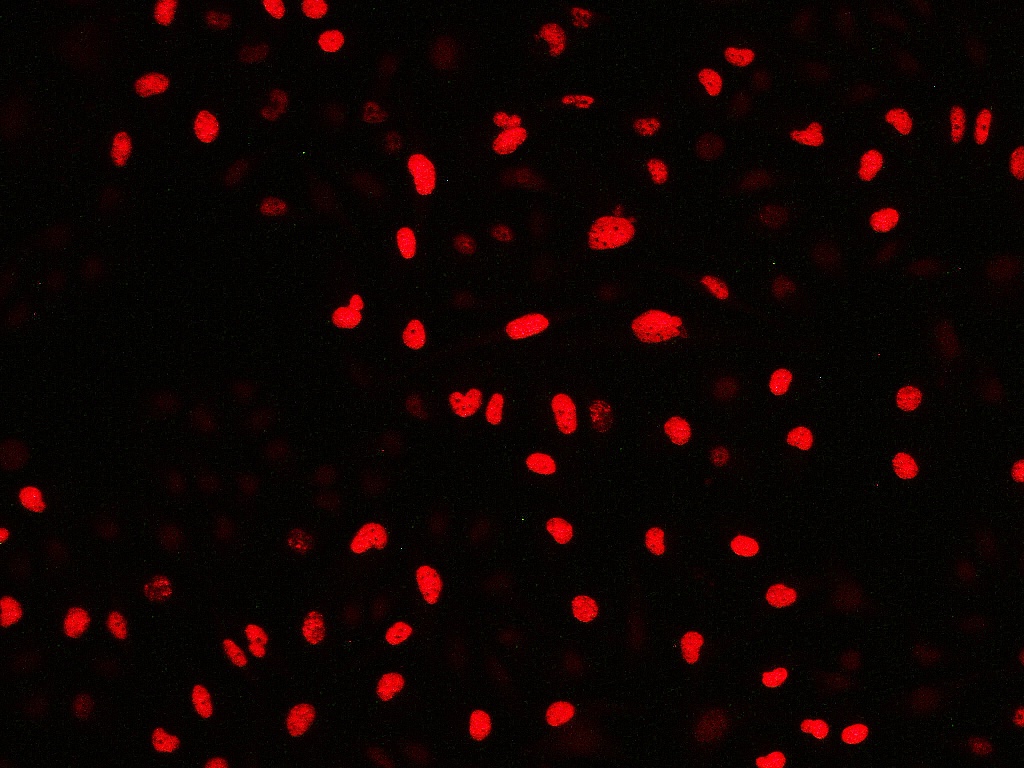

Supplement: Supplementary file 1 [file Data_Sheet_1.ZIP › 975640_SupMaterial/975640_SupMaterial/raw data frontiers/Figure 1/D/Linc00452-OE+Ox-LDL 200-2.jpg]

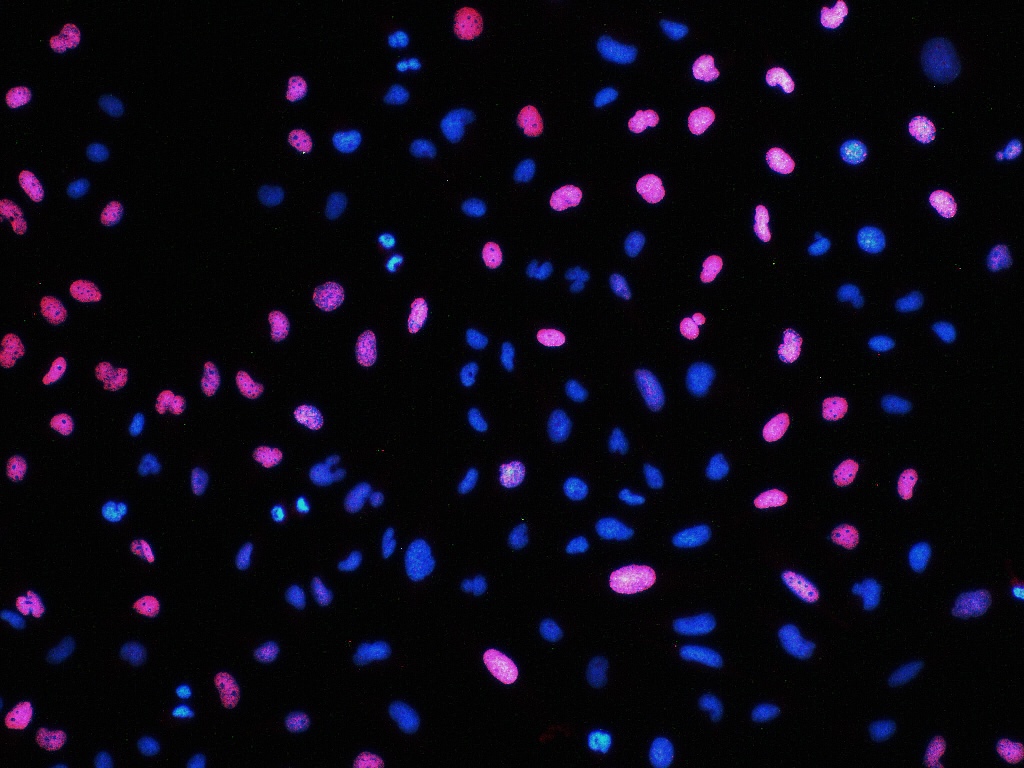

Supplement: Supplementary file 1 [file Data_Sheet_1.ZIP › 975640_SupMaterial/975640_SupMaterial/raw data frontiers/Figure 1/D/Ox-LDL 200-1+2.JPG]

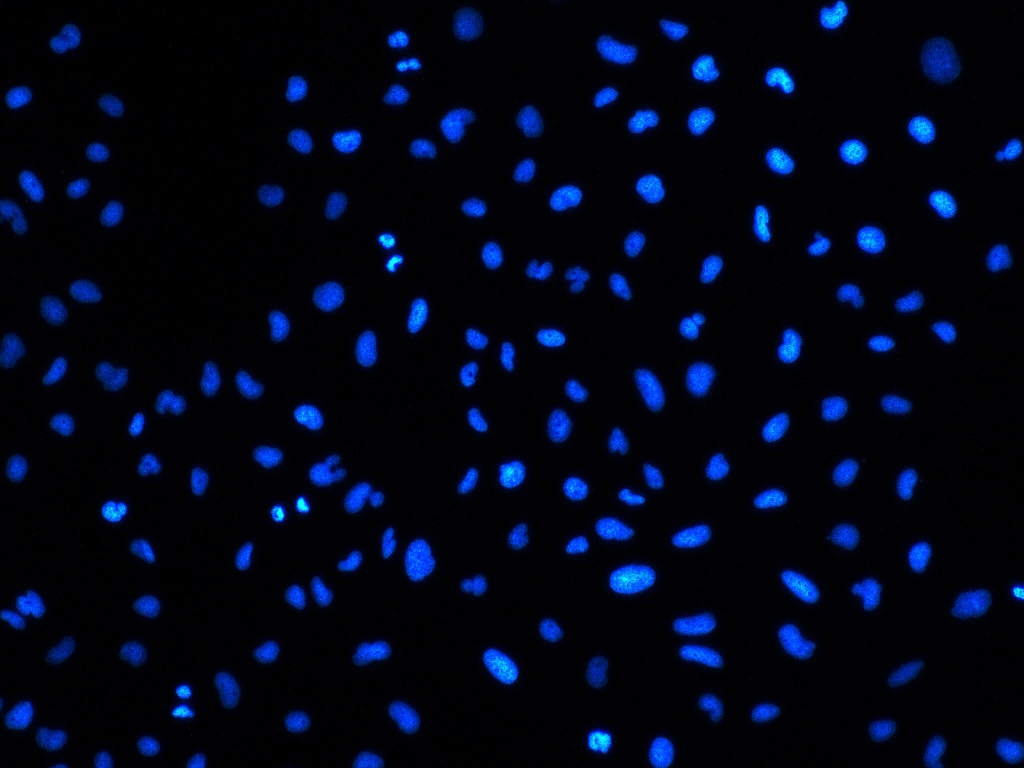

Supplement: Supplementary file 1 [file Data_Sheet_1.ZIP › 975640_SupMaterial/975640_SupMaterial/raw data frontiers/Figure 1/D/Ox-LDL 200-1.jpg]

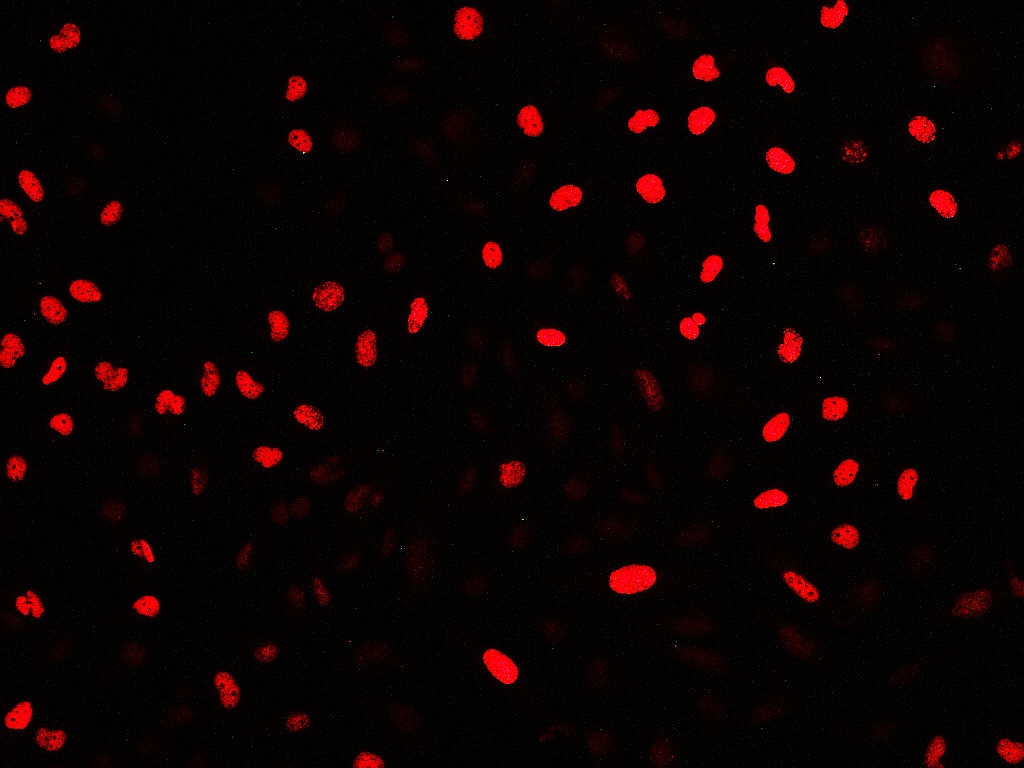

Supplement: Supplementary file 1 [file Data_Sheet_1.ZIP › 975640_SupMaterial/975640_SupMaterial/raw data frontiers/Figure 1/D/Ox-LDL 200-2.jpg]

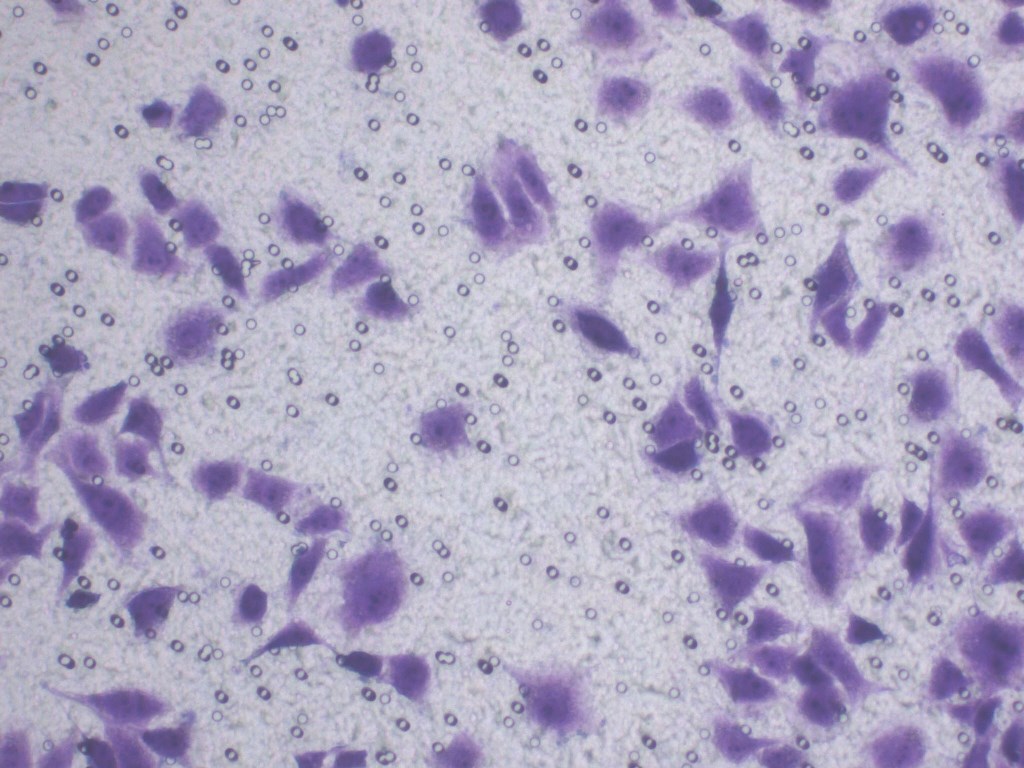

Supplement: Supplementary file 1 [file Data_Sheet_1.ZIP › 975640_SupMaterial/975640_SupMaterial/raw data frontiers/Figure 2/B/transwell/Blank.jpg]

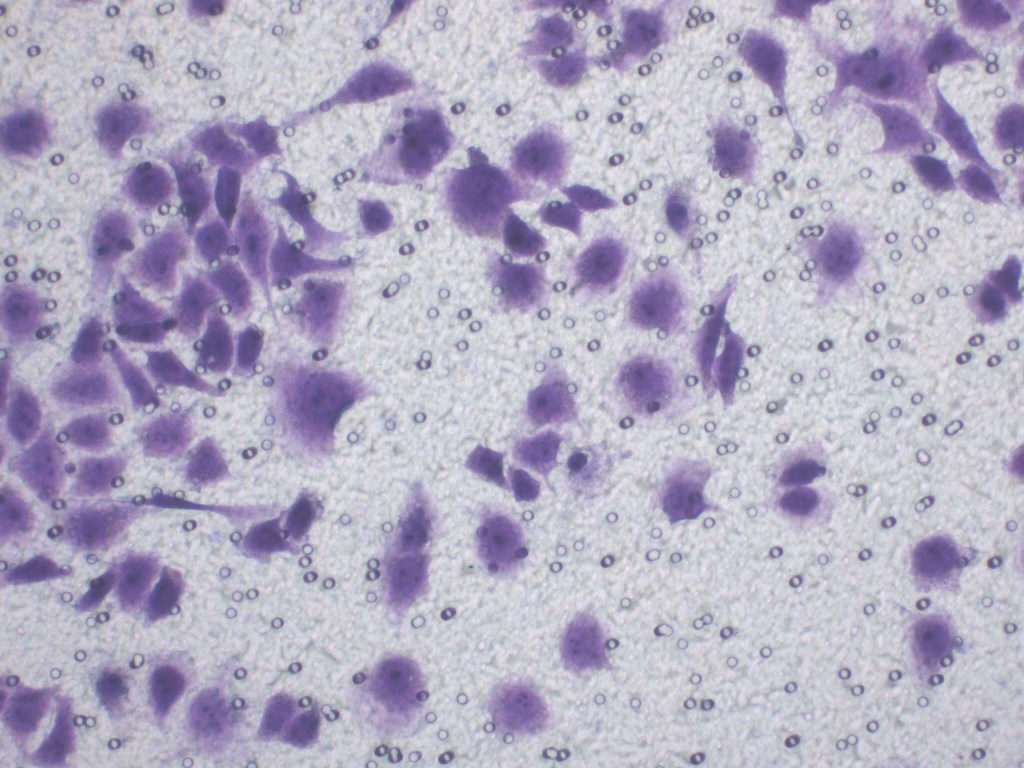

Supplement: Supplementary file 1 [file Data_Sheet_1.ZIP › 975640_SupMaterial/975640_SupMaterial/raw data frontiers/Figure 2/B/transwell/OE.jpg]

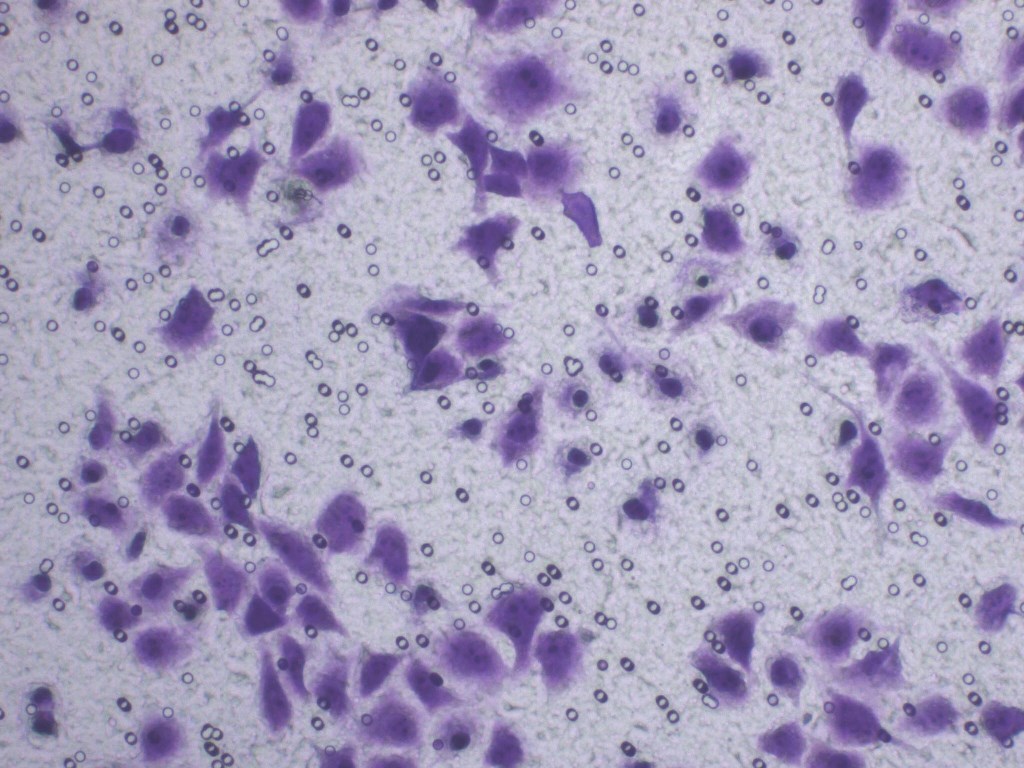

Supplement: Supplementary file 1 [file Data_Sheet_1.ZIP › 975640_SupMaterial/975640_SupMaterial/raw data frontiers/Figure 2/B/transwell/oxLDL + OE.jpg]

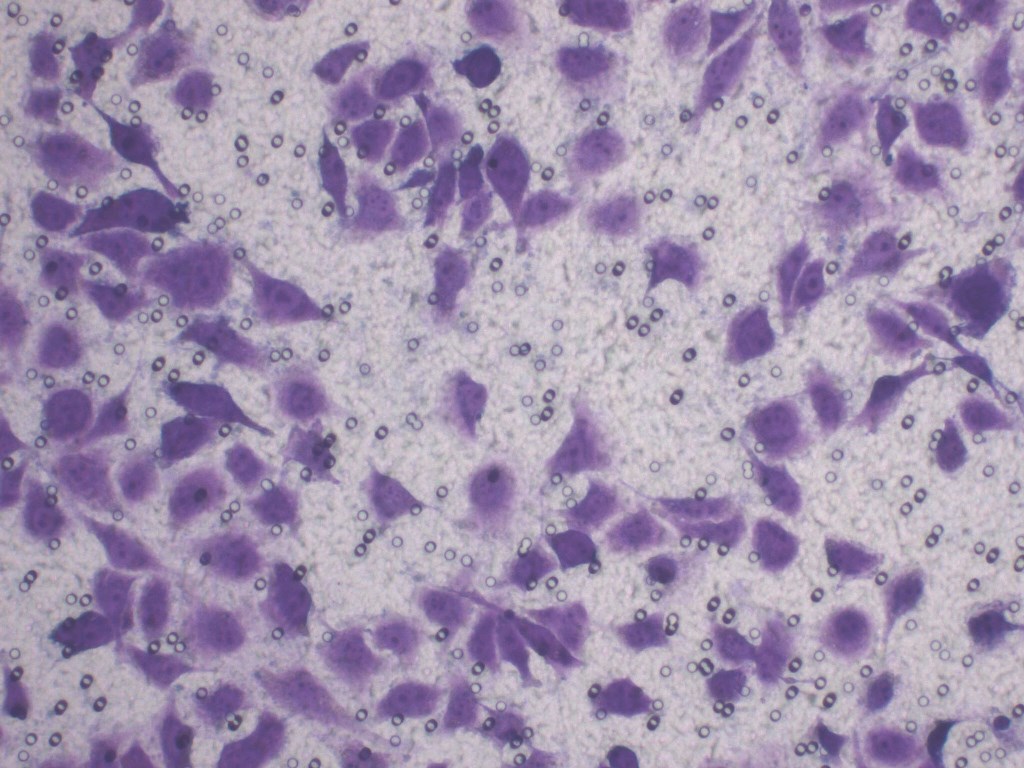

Supplement: Supplementary file 1 [file Data_Sheet_1.ZIP › 975640_SupMaterial/975640_SupMaterial/raw data frontiers/Figure 2/B/transwell/oxLDL.jpg]

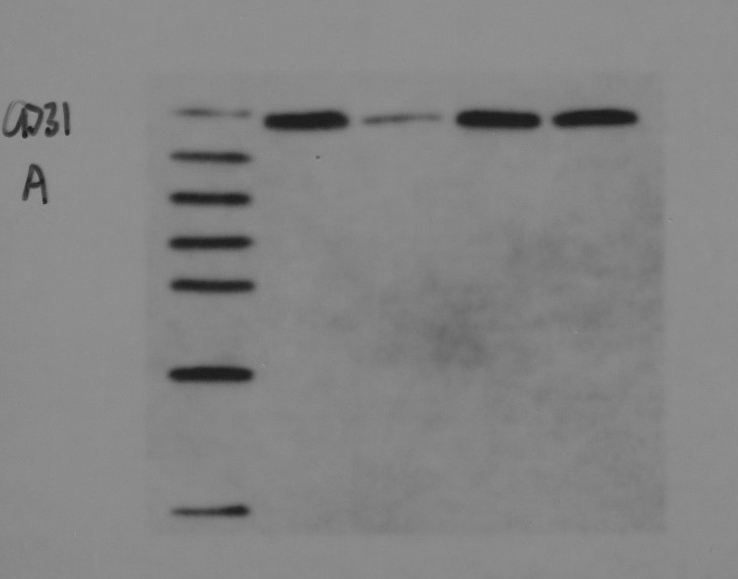

Supplement: Supplementary file 1 [file Data_Sheet_1.ZIP › 975640_SupMaterial/975640_SupMaterial/raw data frontiers/Figure 2/C/CD31-A.jpg]

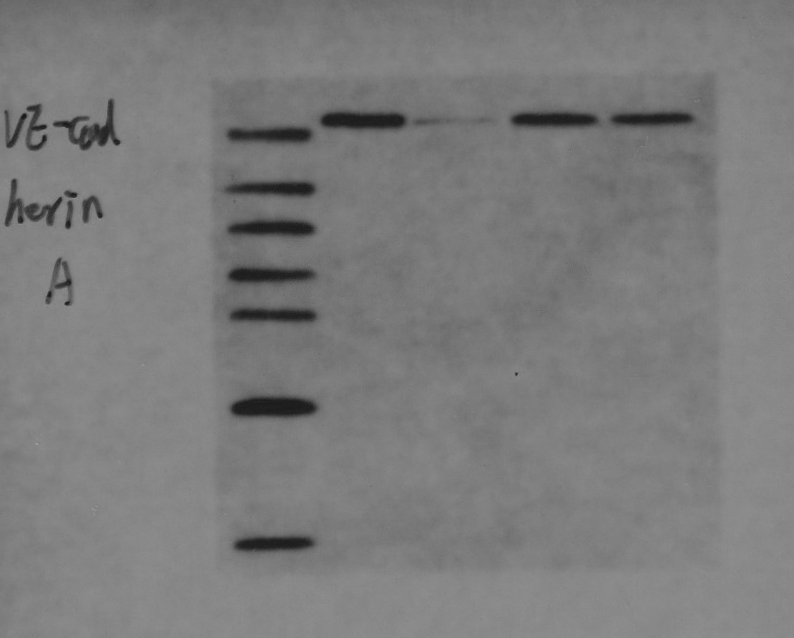

Supplement: Supplementary file 1 [file Data_Sheet_1.ZIP › 975640_SupMaterial/975640_SupMaterial/raw data frontiers/Figure 2/C/VE-cadherin-A.jpg]

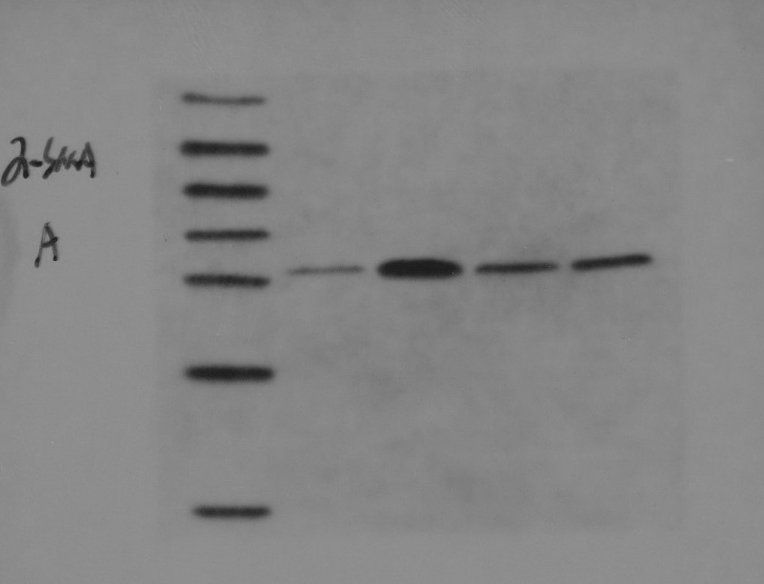

Supplement: Supplementary file 1 [file Data_Sheet_1.ZIP › 975640_SupMaterial/975640_SupMaterial/raw data frontiers/Figure 2/C/α-SMA-A.jpg]

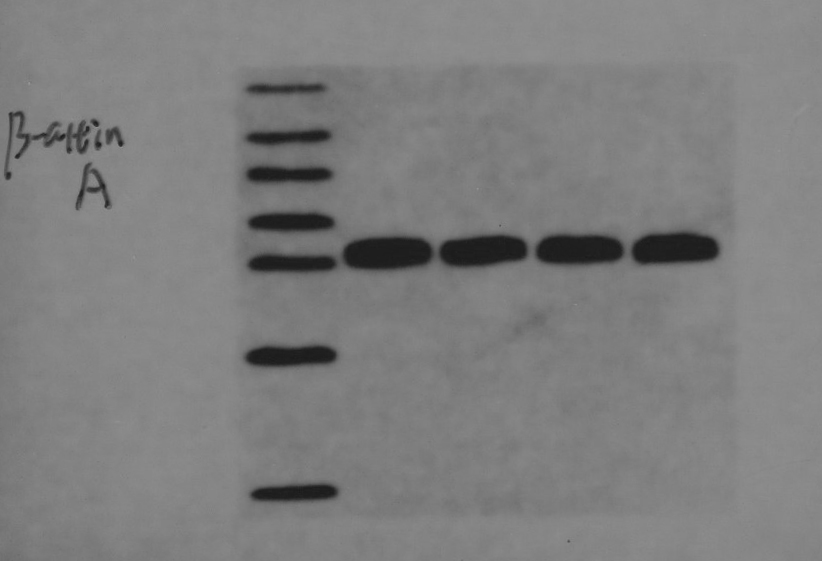

Supplement: Supplementary file 1 [file Data_Sheet_1.ZIP › 975640_SupMaterial/975640_SupMaterial/raw data frontiers/Figure 2/C/β-actin-A.jpg]

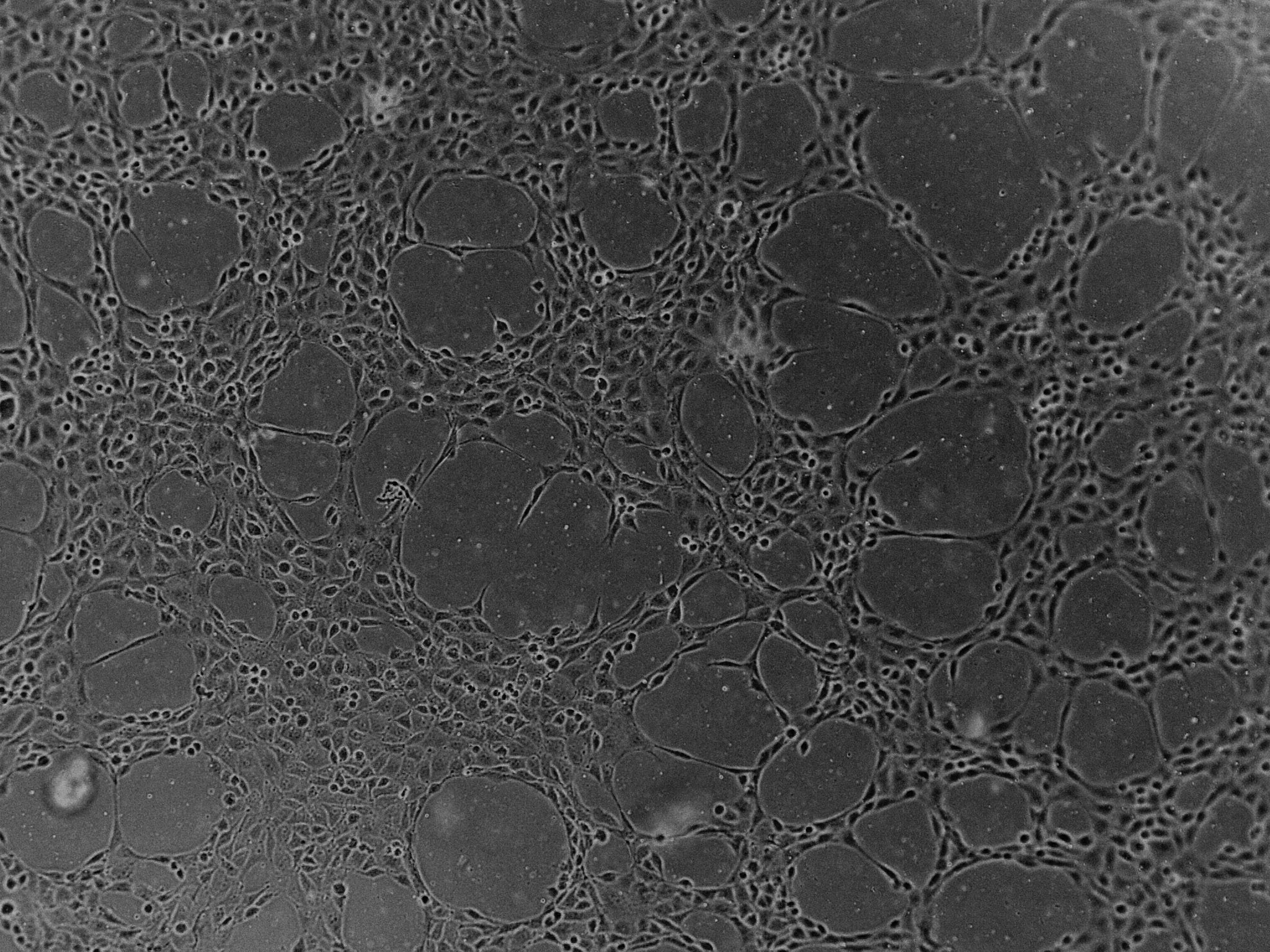

Supplement: Supplementary file 1 [file Data_Sheet_1.ZIP › 975640_SupMaterial/975640_SupMaterial/raw data frontiers/Figure 3/A/Control.jpg]

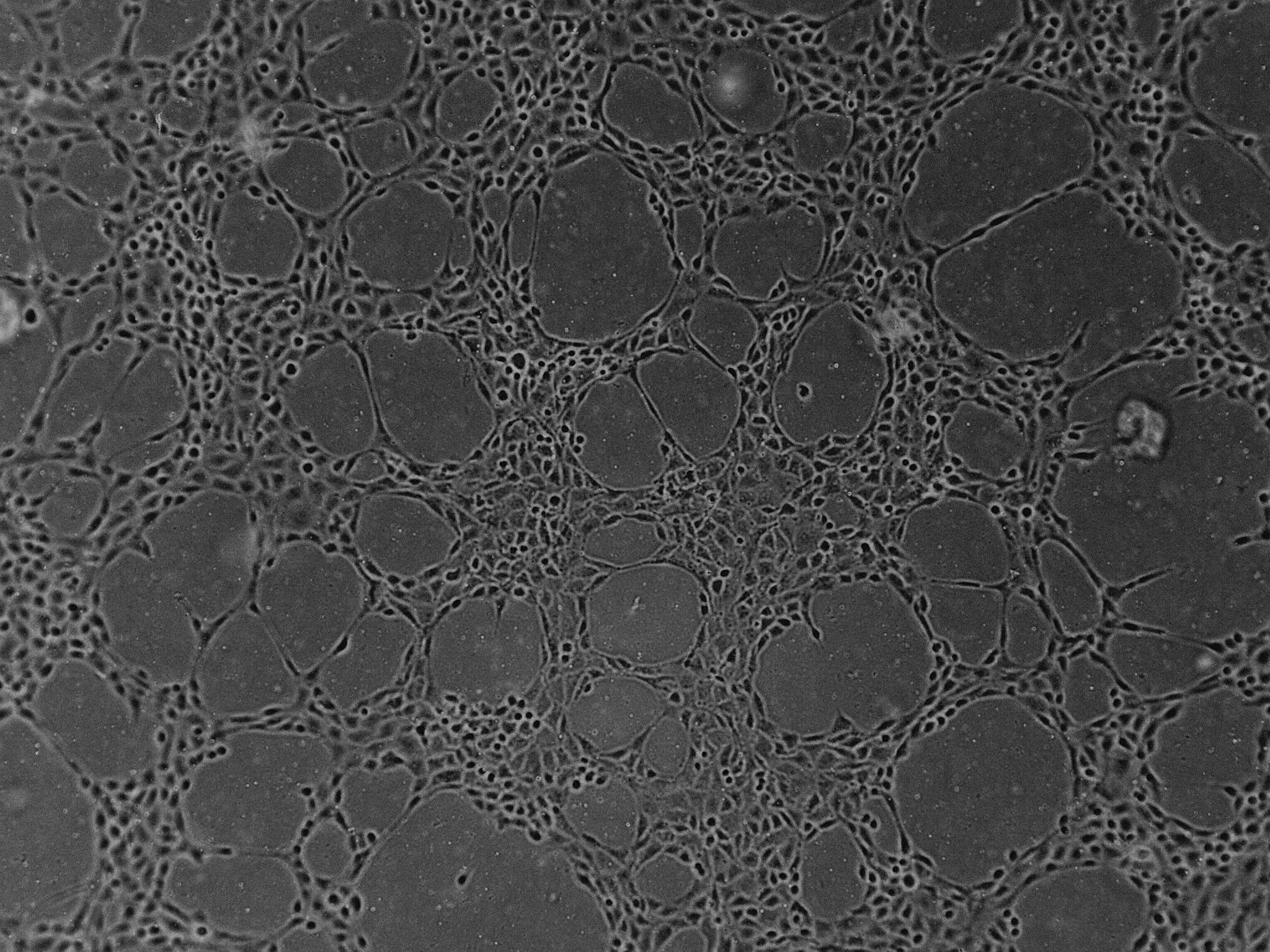

Supplement: Supplementary file 1 [file Data_Sheet_1.ZIP › 975640_SupMaterial/975640_SupMaterial/raw data frontiers/Figure 3/A/OE.jpg]

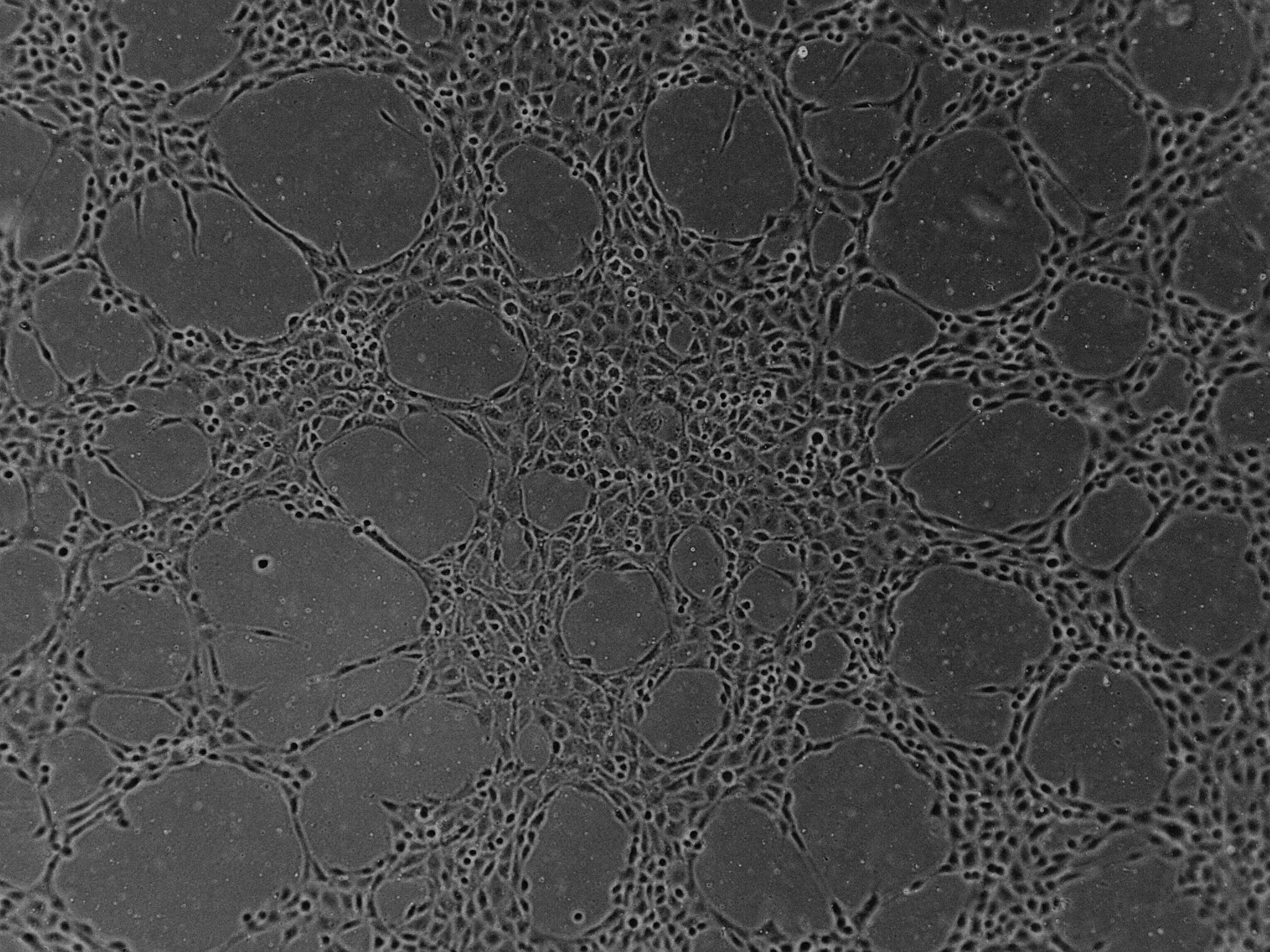

Supplement: Supplementary file 1 [file Data_Sheet_1.ZIP › 975640_SupMaterial/975640_SupMaterial/raw data frontiers/Figure 3/A/ox-LDL + OE.jpg]

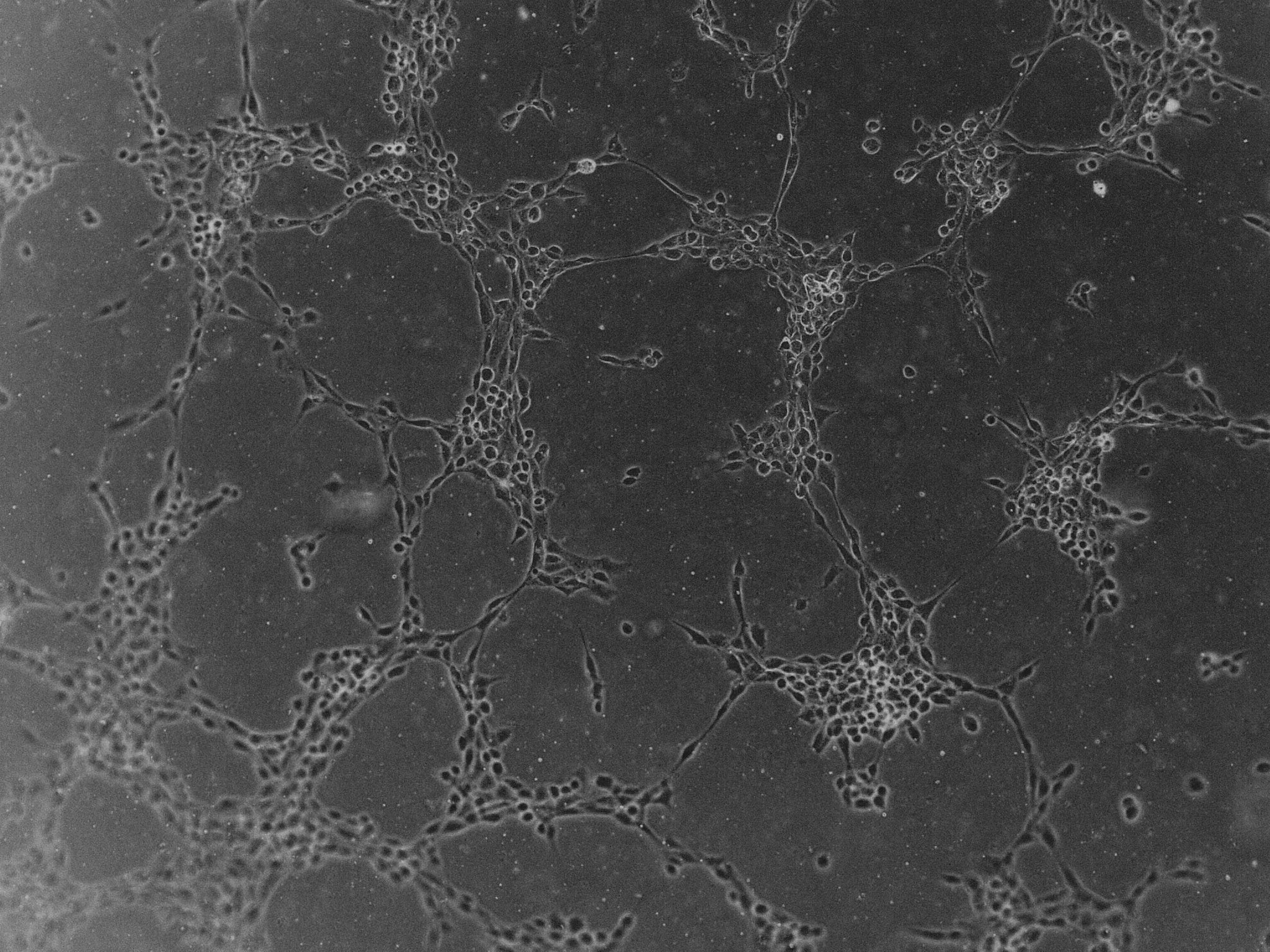

Supplement: Supplementary file 1 [file Data_Sheet_1.ZIP › 975640_SupMaterial/975640_SupMaterial/raw data frontiers/Figure 3/A/ox-LDL.jpg]

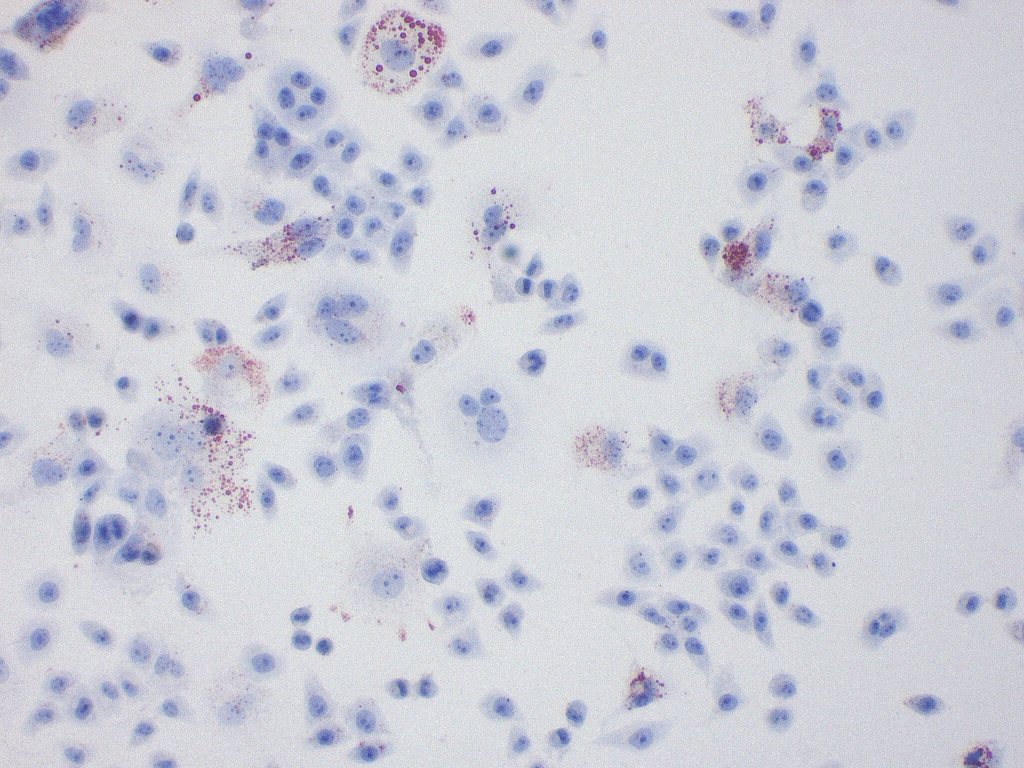

Supplement: Supplementary file 1 [file Data_Sheet_1.ZIP › 975640_SupMaterial/975640_SupMaterial/raw data frontiers/Figure 3/B/Control.jpg]

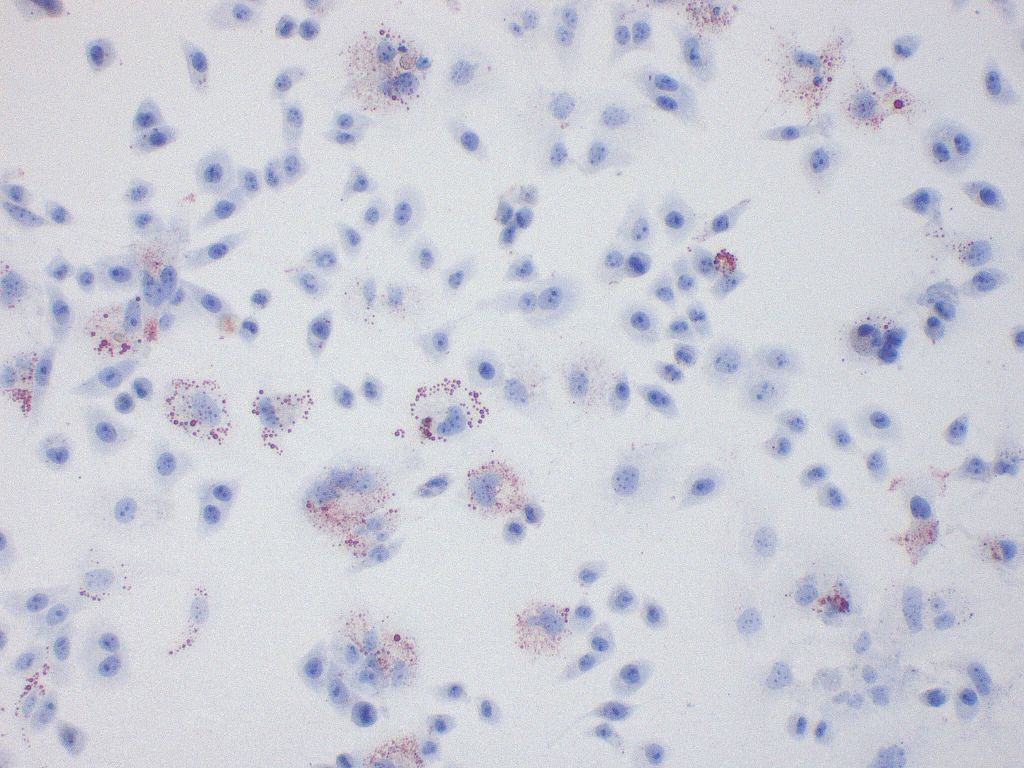

Supplement: Supplementary file 1 [file Data_Sheet_1.ZIP › 975640_SupMaterial/975640_SupMaterial/raw data frontiers/Figure 3/B/OE.jpg]

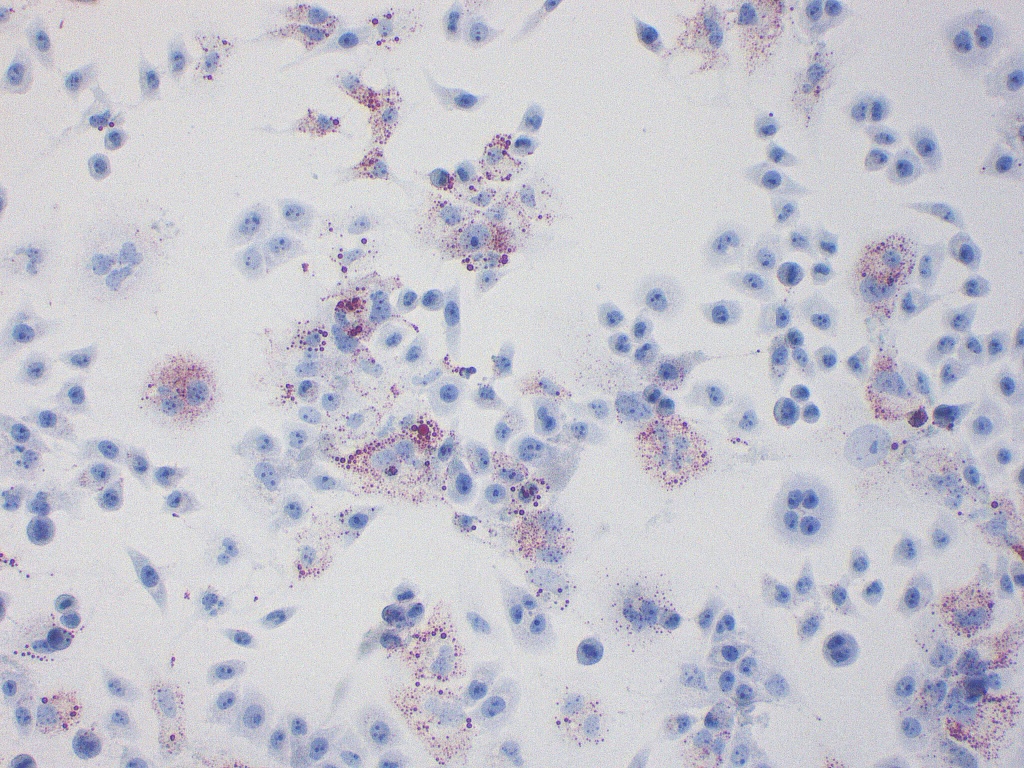

Supplement: Supplementary file 1 [file Data_Sheet_1.ZIP › 975640_SupMaterial/975640_SupMaterial/raw data frontiers/Figure 3/B/ox-LDL + OE.jpg]

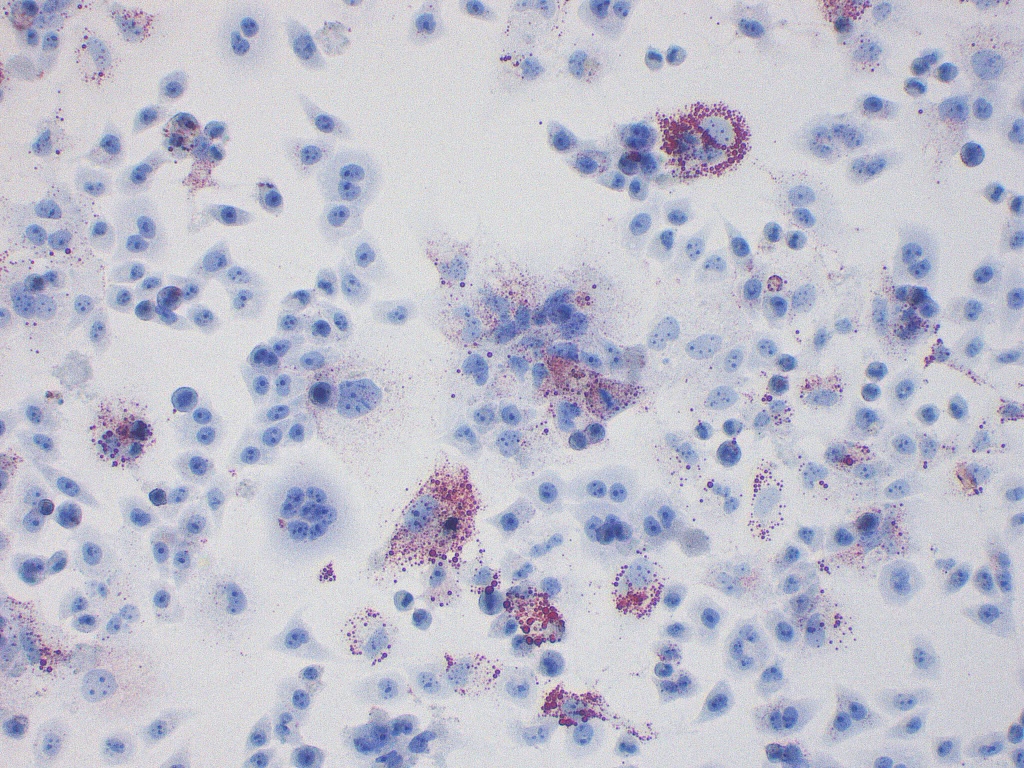

Supplement: Supplementary file 1 [file Data_Sheet_1.ZIP › 975640_SupMaterial/975640_SupMaterial/raw data frontiers/Figure 3/B/ox-LDL.jpg]

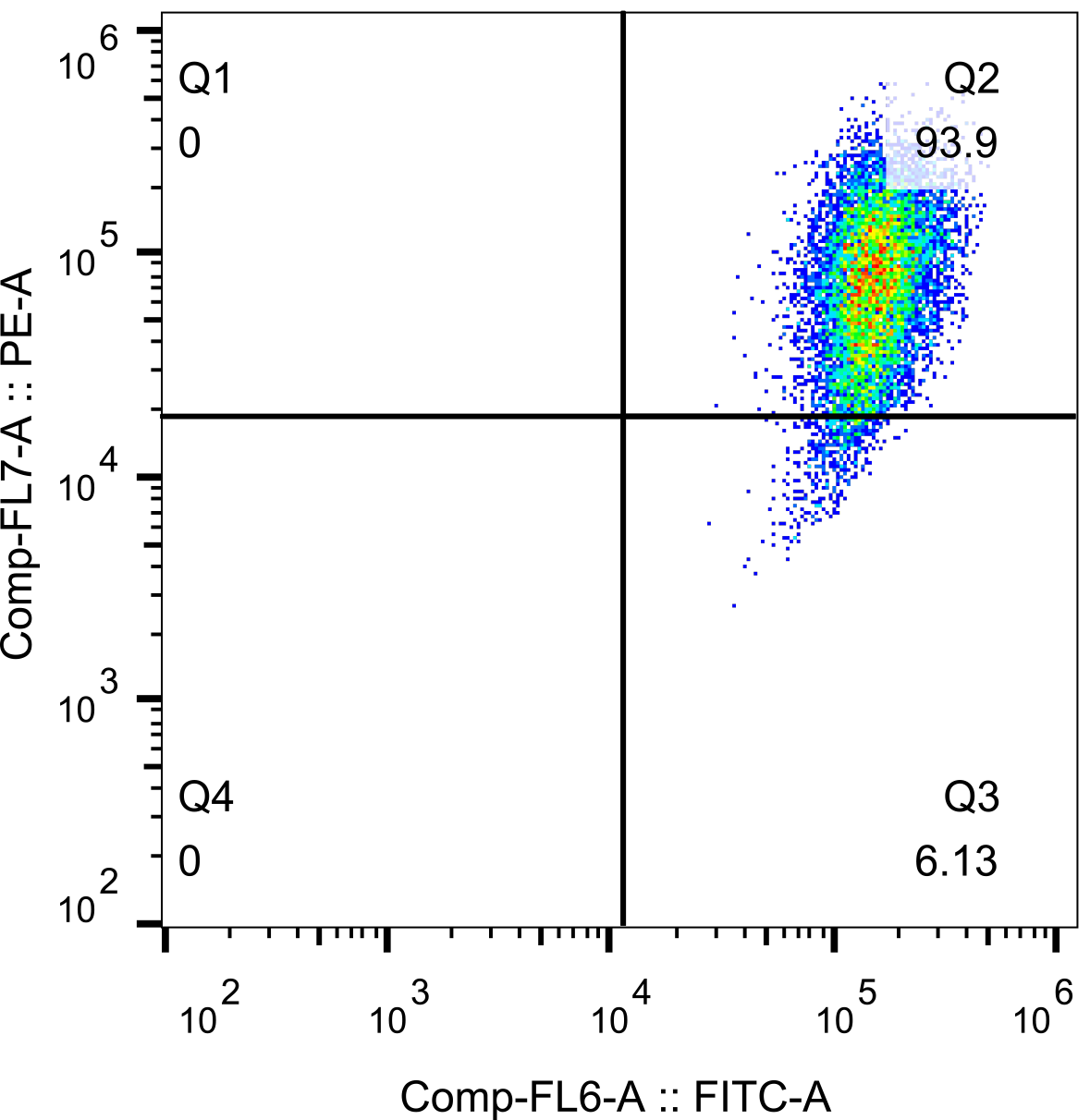

Supplement: Supplementary file 1 [file Data_Sheet_1.ZIP › 975640_SupMaterial/975640_SupMaterial/raw data frontiers/Figure 3/C/01-1.tif]

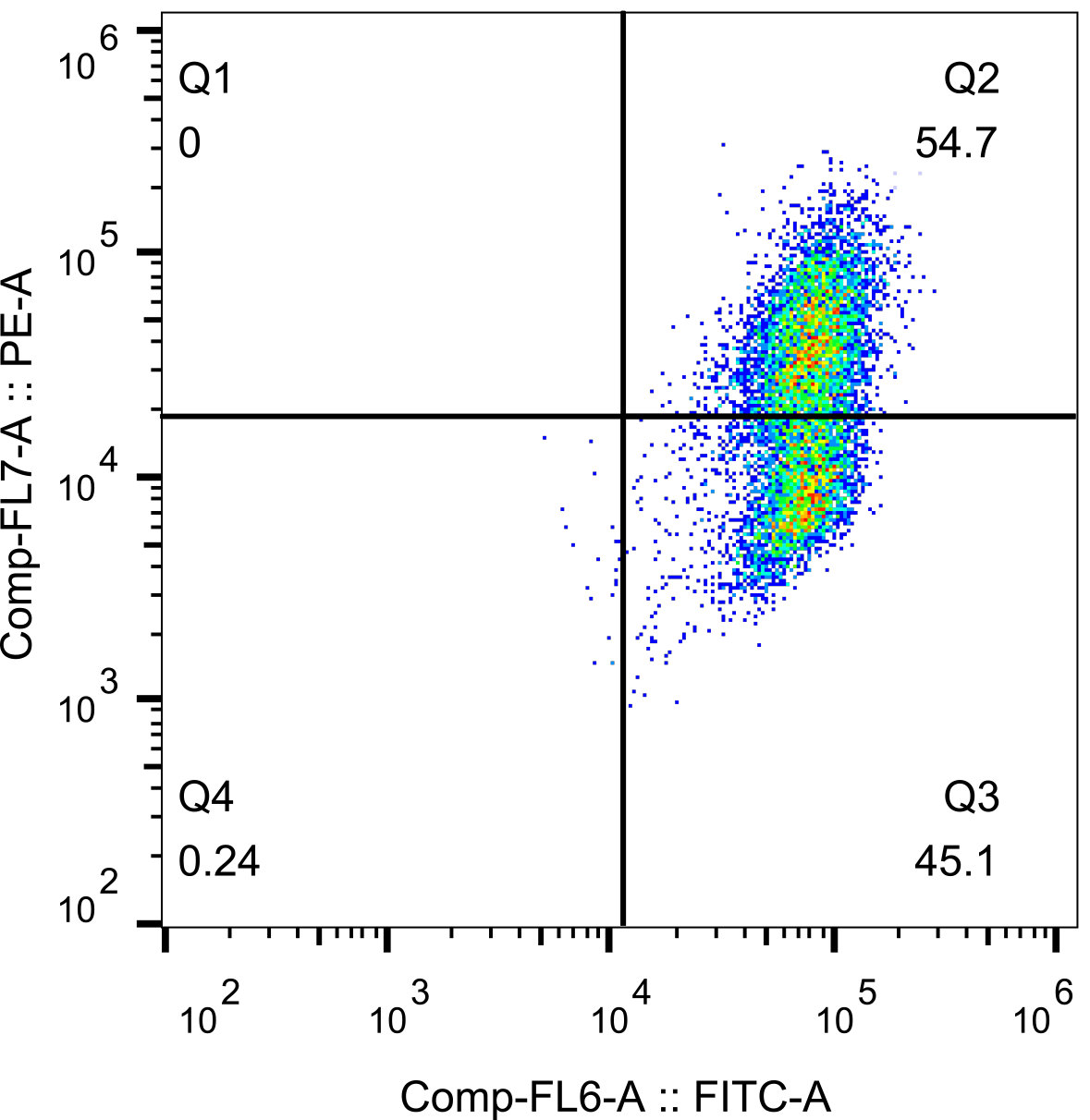

Supplement: Supplementary file 1 [file Data_Sheet_1.ZIP › 975640_SupMaterial/975640_SupMaterial/raw data frontiers/Figure 3/C/02-1.tif]

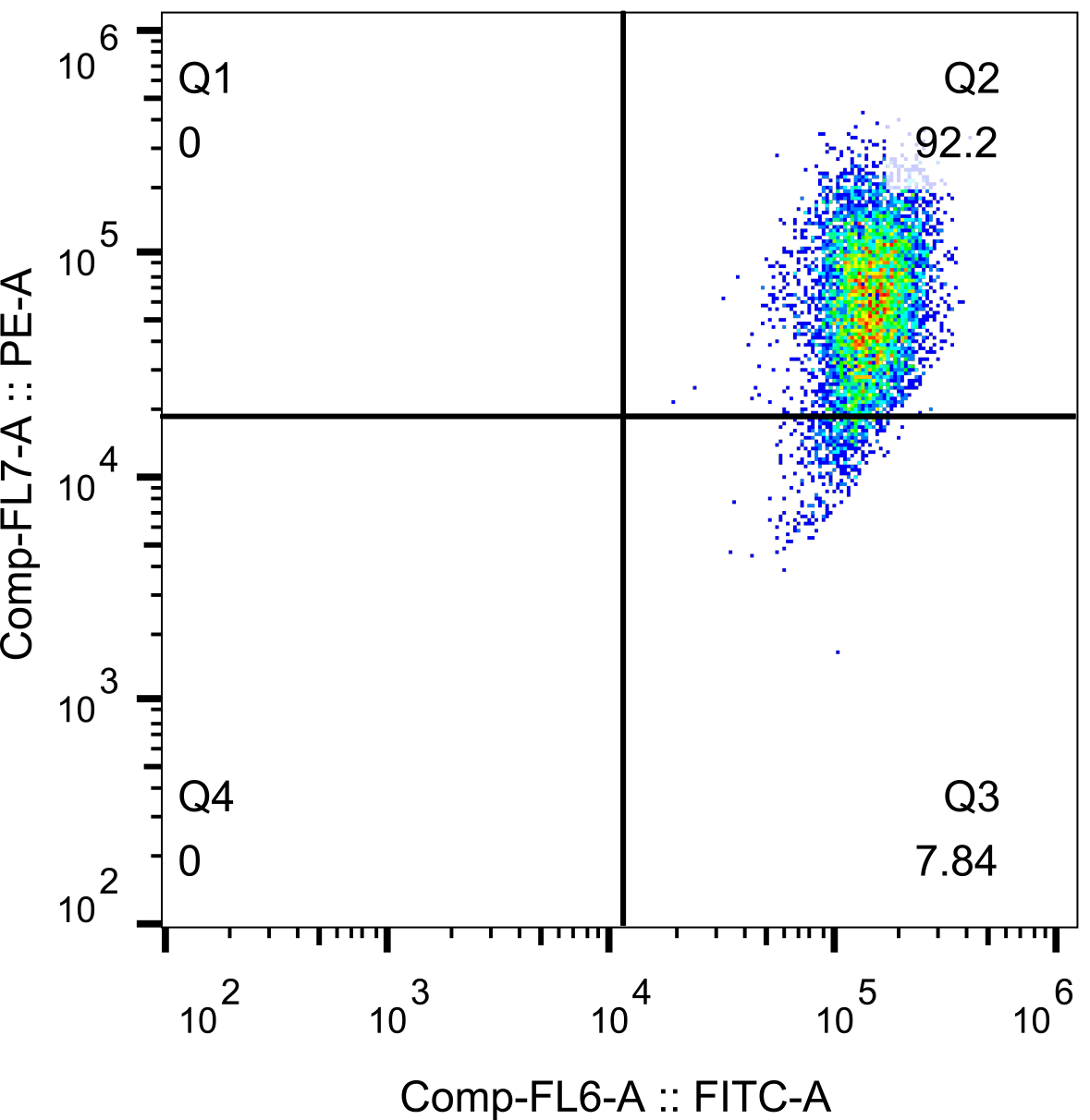

Supplement: Supplementary file 1 [file Data_Sheet_1.ZIP › 975640_SupMaterial/975640_SupMaterial/raw data frontiers/Figure 3/C/03-1.tif]

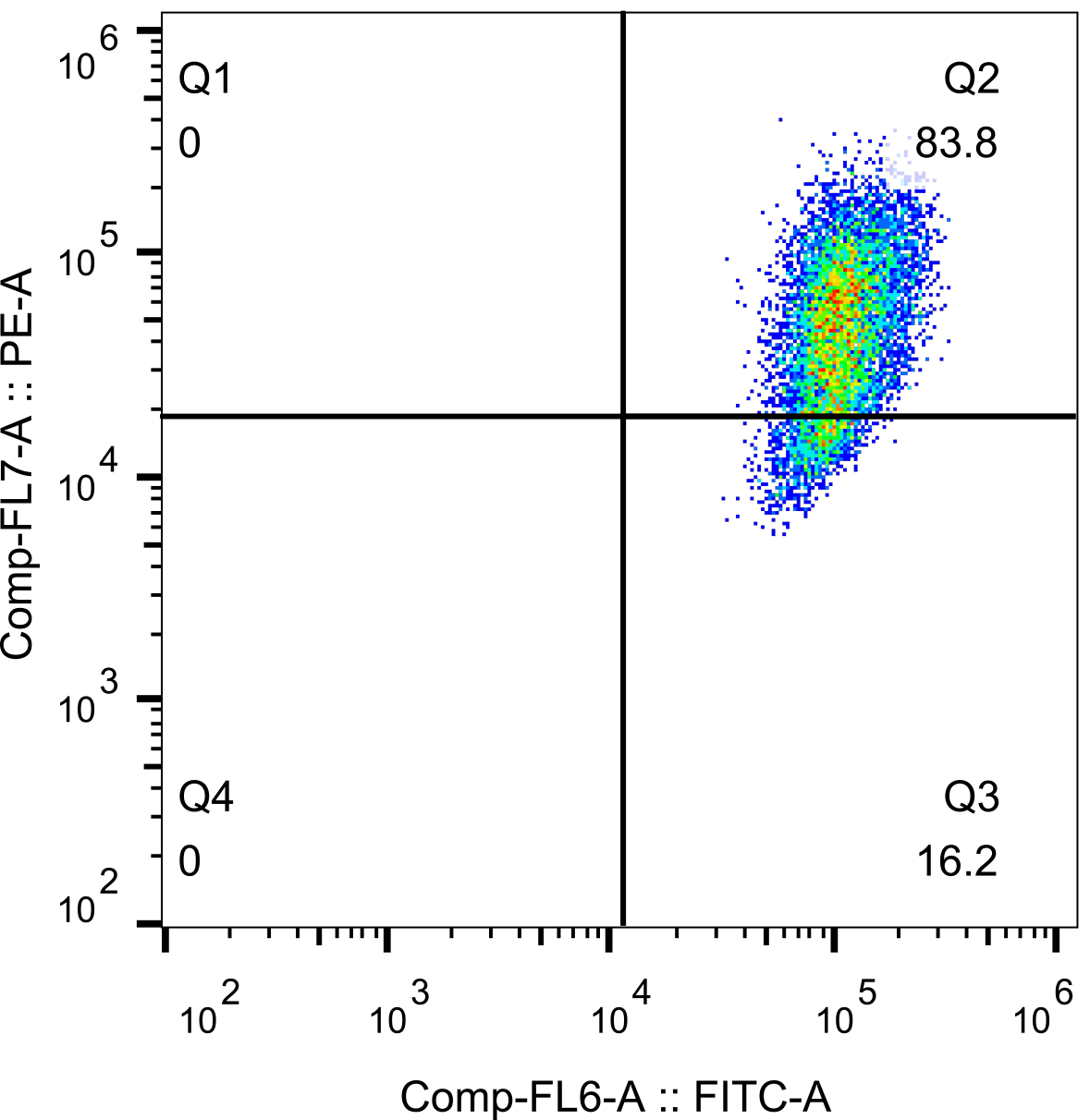

Supplement: Supplementary file 1 [file Data_Sheet_1.ZIP › 975640_SupMaterial/975640_SupMaterial/raw data frontiers/Figure 3/C/04-1.tif]

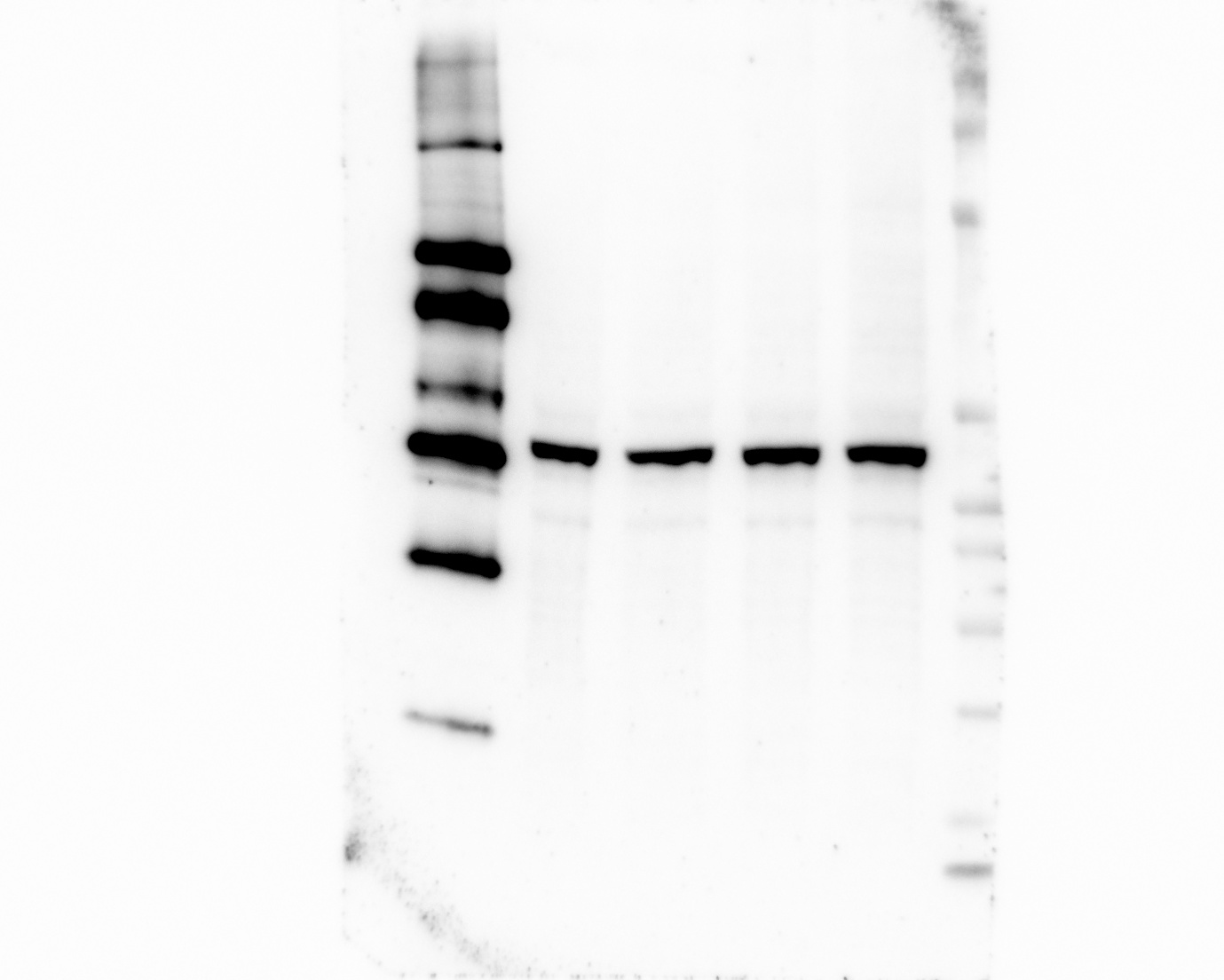

Supplement: Supplementary file 1 [file Data_Sheet_1.ZIP › 975640_SupMaterial/975640_SupMaterial/raw data frontiers/Figure 4/F, G, H/b-actin (Fig.4F).jpg]

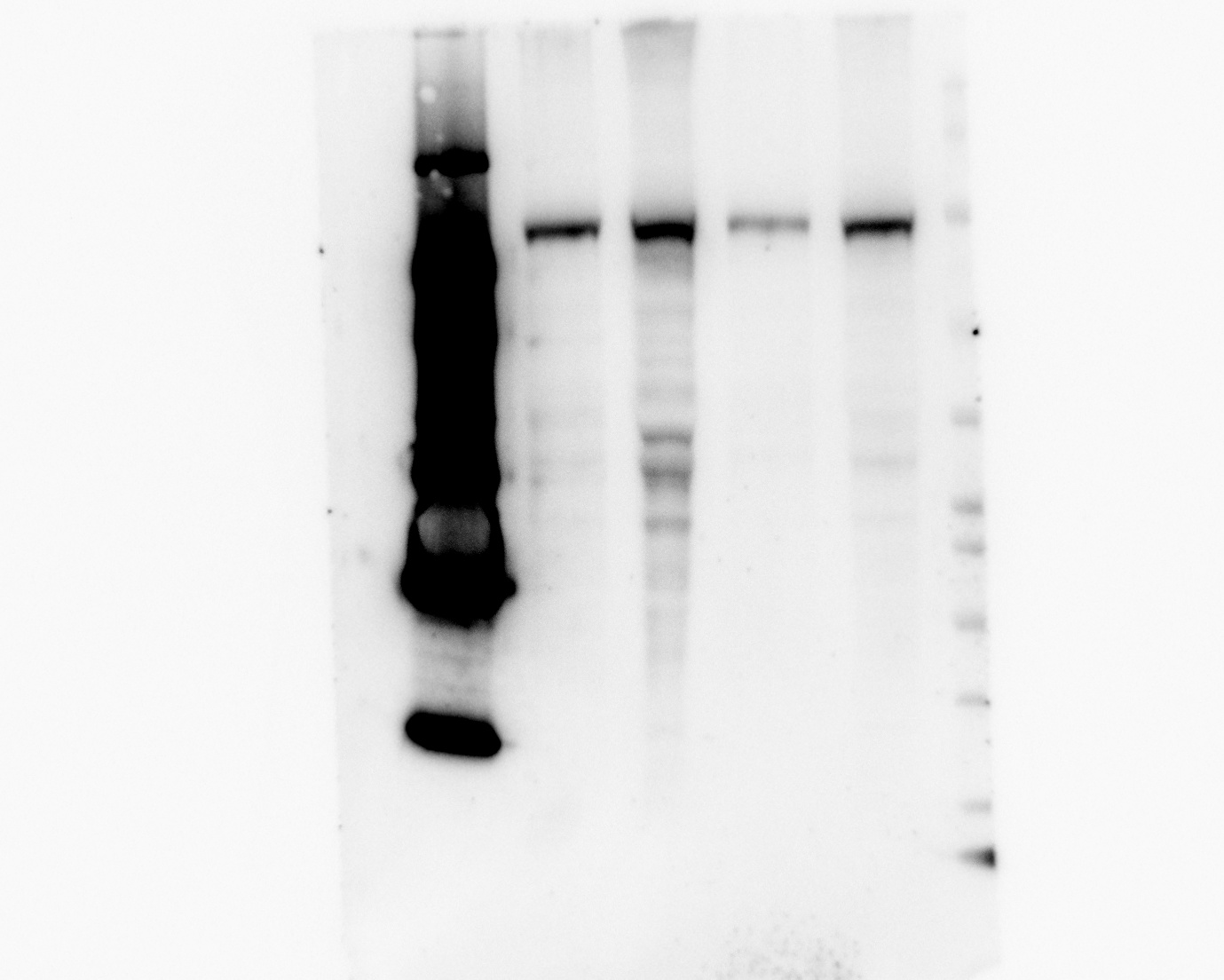

Supplement: Supplementary file 1 [file Data_Sheet_1.ZIP › 975640_SupMaterial/975640_SupMaterial/raw data frontiers/Figure 4/F, G, H/b-catenin-2-1 fig. 4f.jpg]

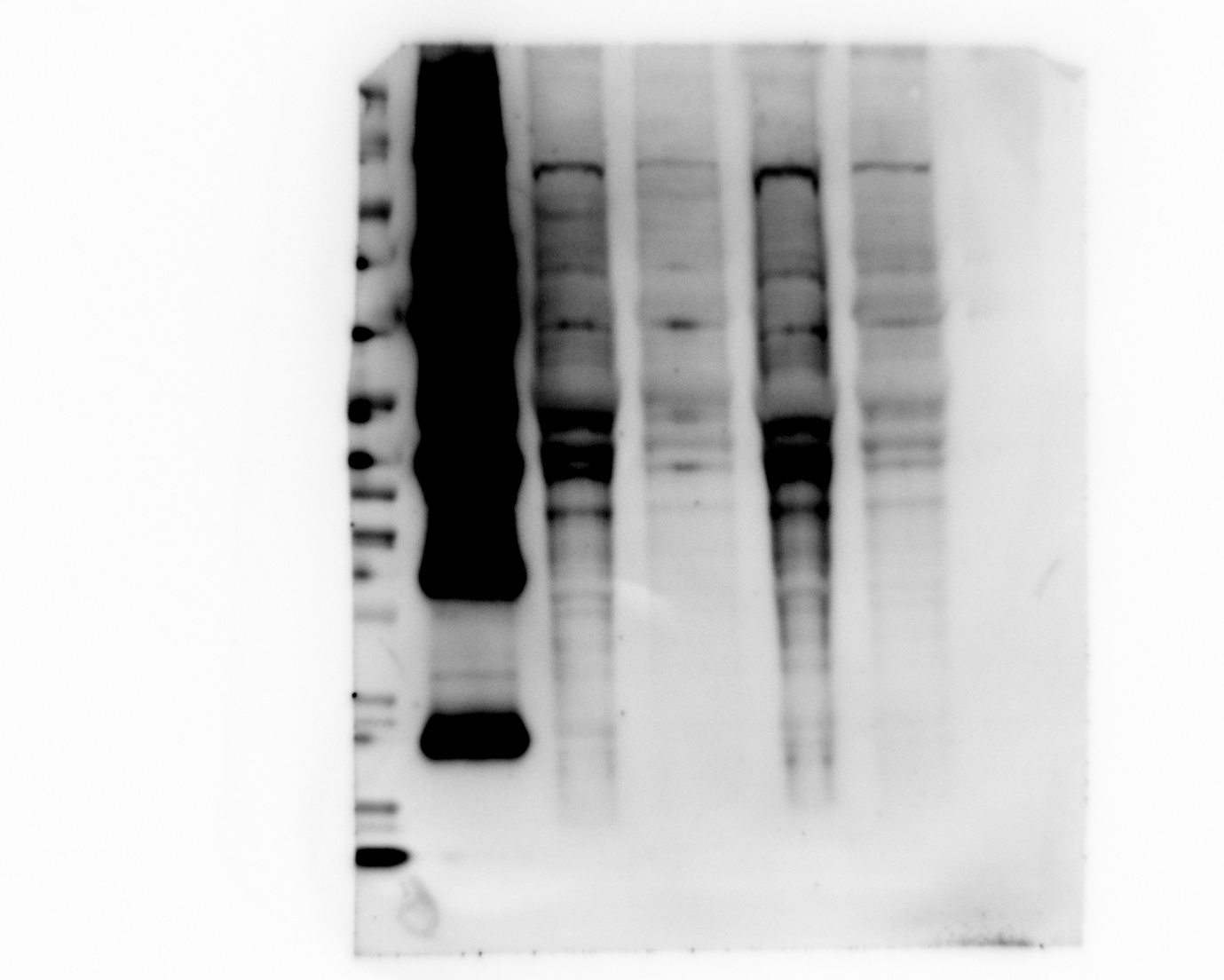

Supplement: Supplementary file 1 [file Data_Sheet_1.ZIP › 975640_SupMaterial/975640_SupMaterial/raw data frontiers/Figure 4/F, G, H/IGF1R (Fig.4F).jpg]

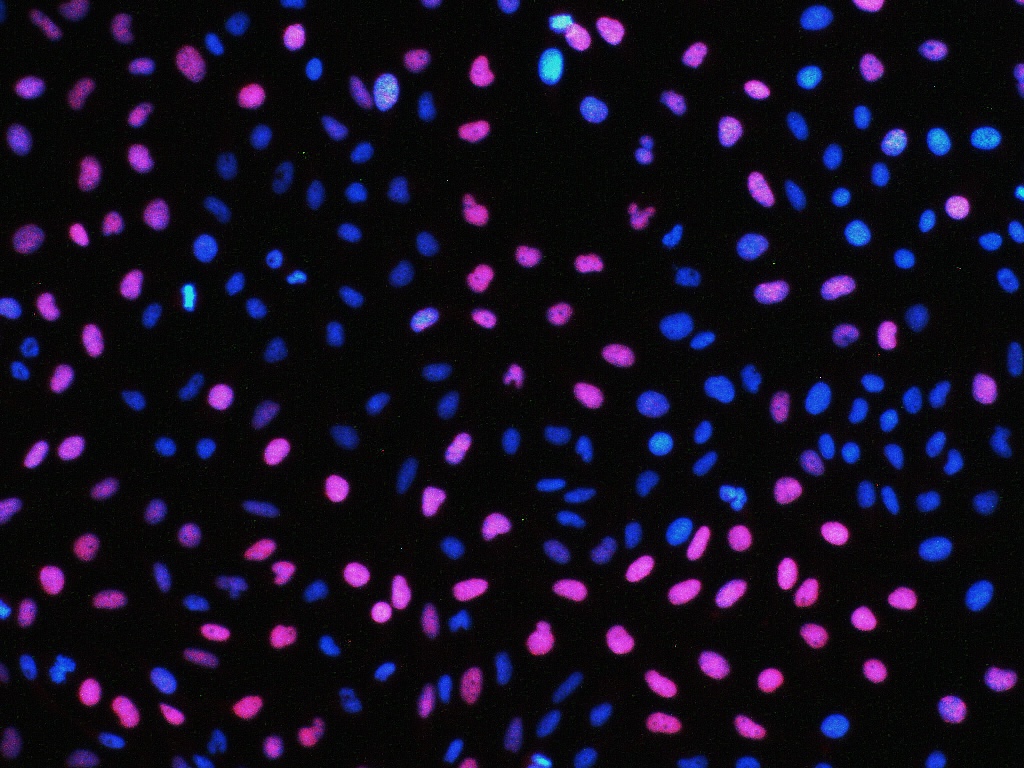

Supplement: Supplementary file 1 [file Data_Sheet_1.ZIP › 975640_SupMaterial/975640_SupMaterial/raw data frontiers/Figure 5/Figure 5A figures/Blank 200-1+2.JPG]

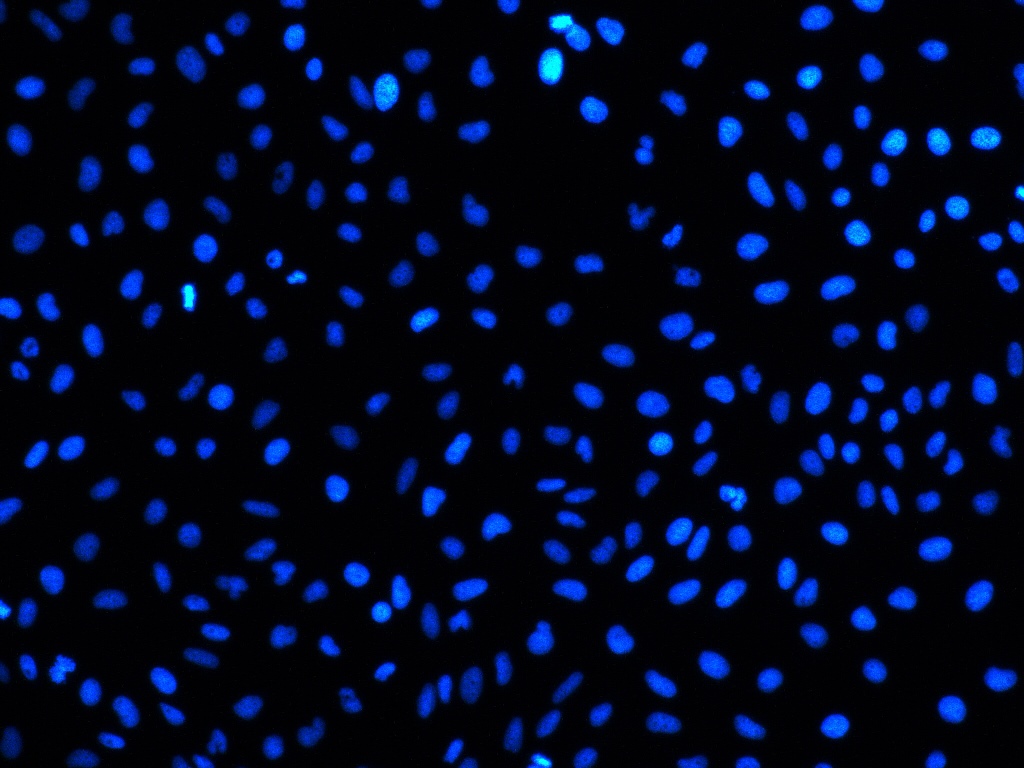

Supplement: Supplementary file 1 [file Data_Sheet_1.ZIP › 975640_SupMaterial/975640_SupMaterial/raw data frontiers/Figure 5/Figure 5A figures/Blank 200-1.jpg]

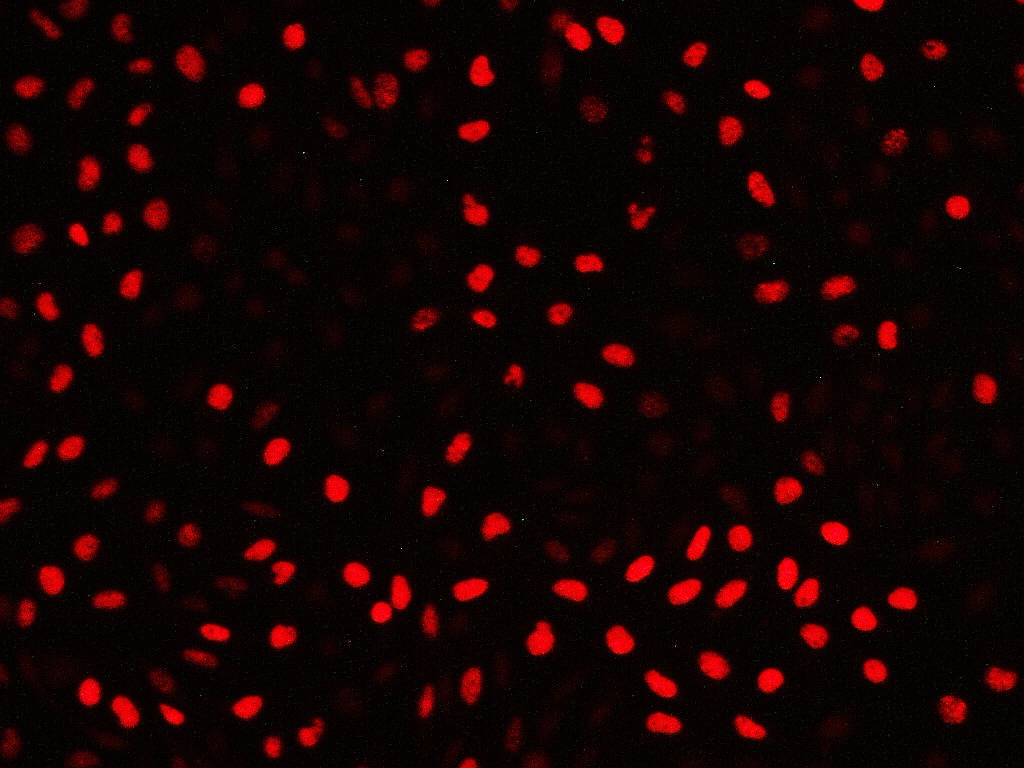

Supplement: Supplementary file 1 [file Data_Sheet_1.ZIP › 975640_SupMaterial/975640_SupMaterial/raw data frontiers/Figure 5/Figure 5A figures/Blank 200-2.jpg]

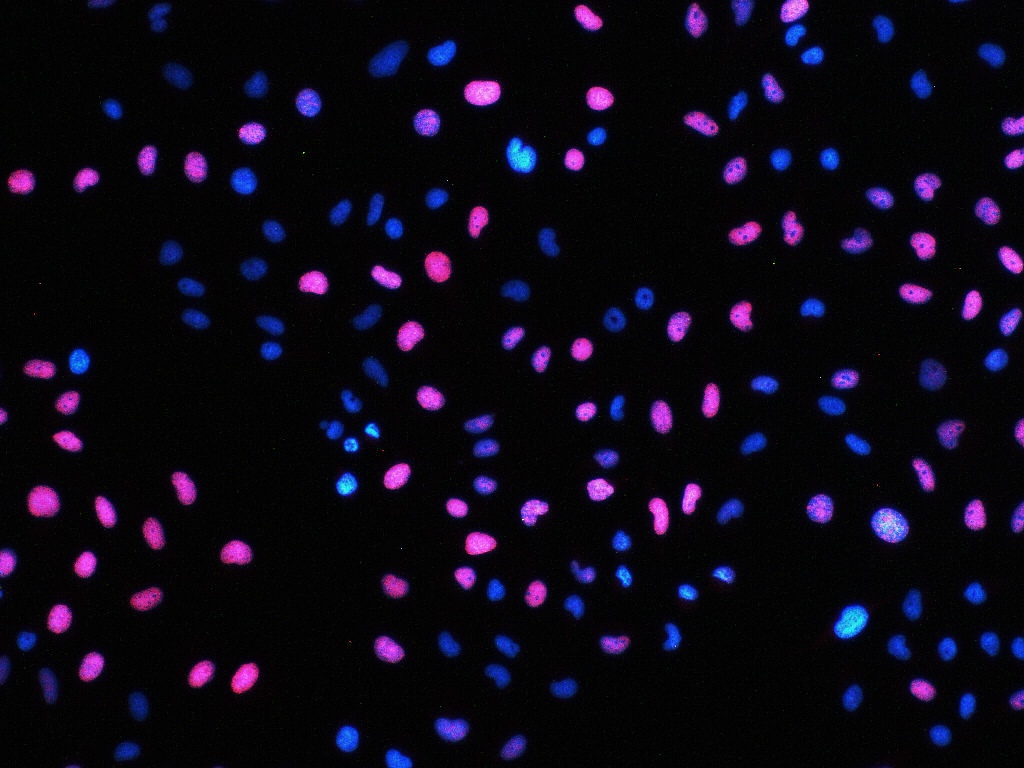

Supplement: Supplementary file 1 [file Data_Sheet_1.ZIP › 975640_SupMaterial/975640_SupMaterial/raw data frontiers/Figure 5/Figure 5A figures/Linc00452-OE+Ox-LDL 200-1+2.JPG]

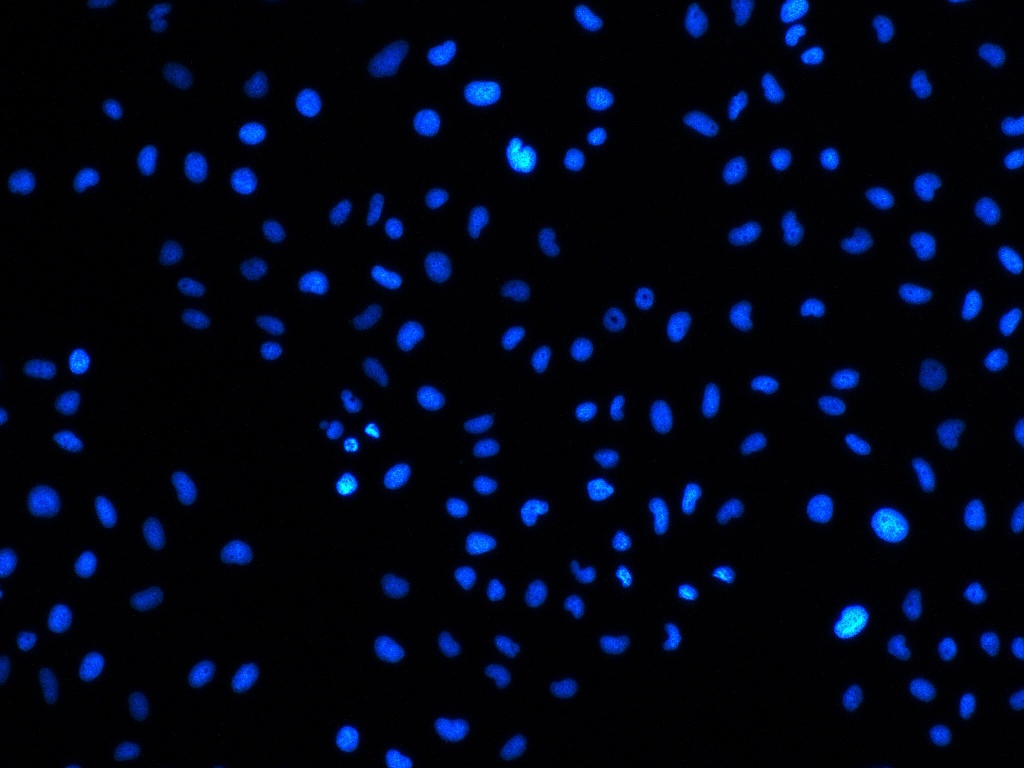

Supplement: Supplementary file 1 [file Data_Sheet_1.ZIP › 975640_SupMaterial/975640_SupMaterial/raw data frontiers/Figure 5/Figure 5A figures/Linc00452-OE+Ox-LDL 200-1.jpg]

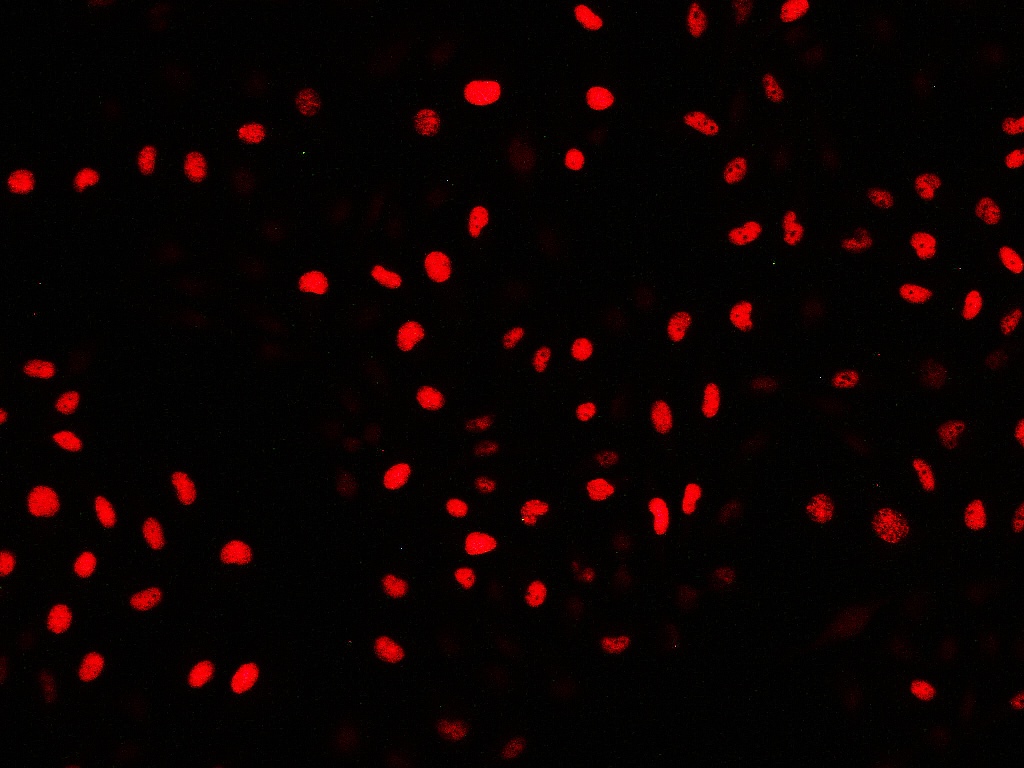

Supplement: Supplementary file 1 [file Data_Sheet_1.ZIP › 975640_SupMaterial/975640_SupMaterial/raw data frontiers/Figure 5/Figure 5A figures/Linc00452-OE+Ox-LDL 200-2.jpg]

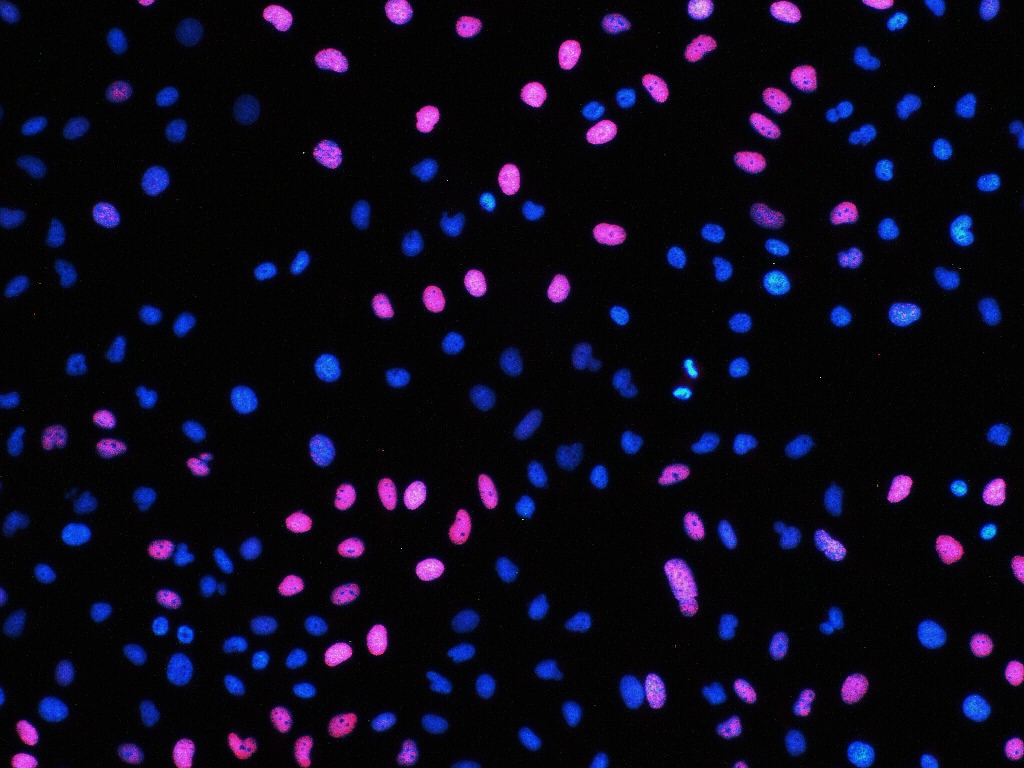

Supplement: Supplementary file 1 [file Data_Sheet_1.ZIP › 975640_SupMaterial/975640_SupMaterial/raw data frontiers/Figure 5/Figure 5A figures/Linc00452-OE+Ox-LDL+IGF1R siRNA 200-3+4.JPG]

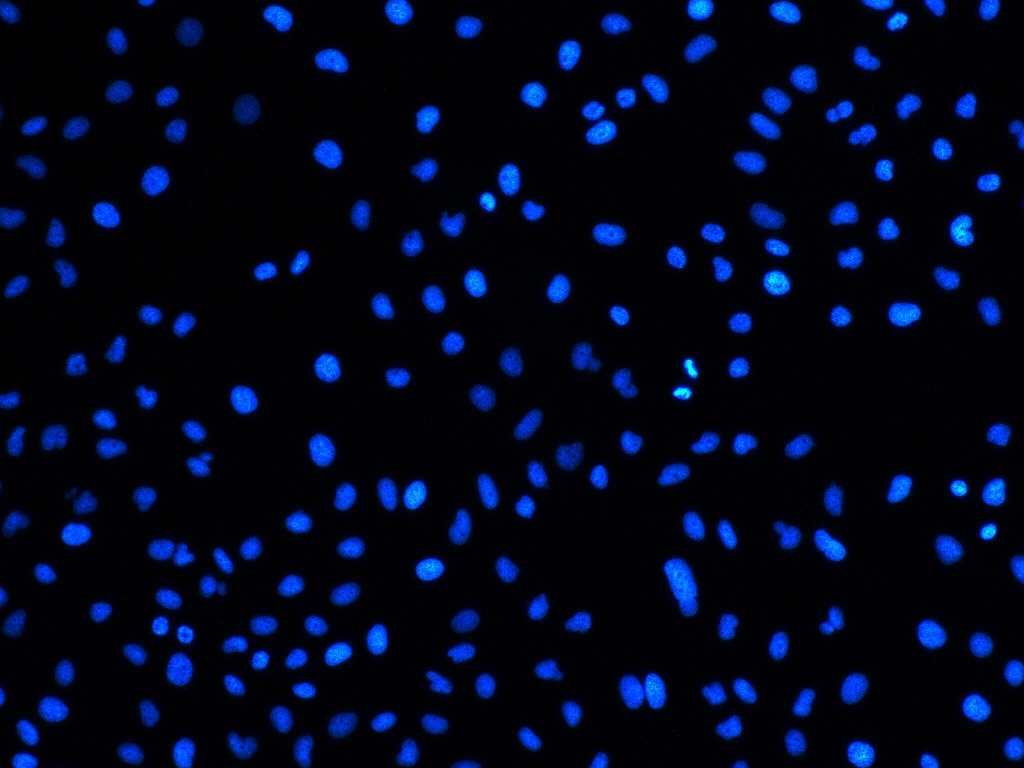

Supplement: Supplementary file 1 [file Data_Sheet_1.ZIP › 975640_SupMaterial/975640_SupMaterial/raw data frontiers/Figure 5/Figure 5A figures/Linc00452-OE+Ox-LDL+IGF1R siRNA 200-3.jpg]

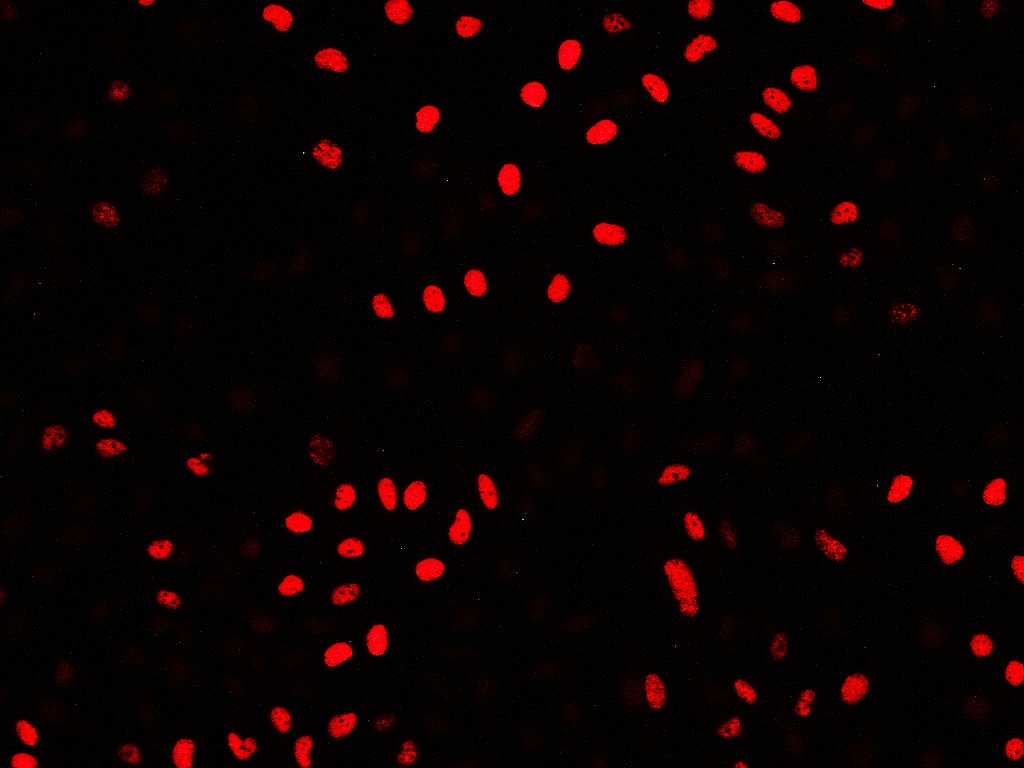

Supplement: Supplementary file 1 [file Data_Sheet_1.ZIP › 975640_SupMaterial/975640_SupMaterial/raw data frontiers/Figure 5/Figure 5A figures/Linc00452-OE+Ox-LDL+IGF1R siRNA 200-4.jpg]

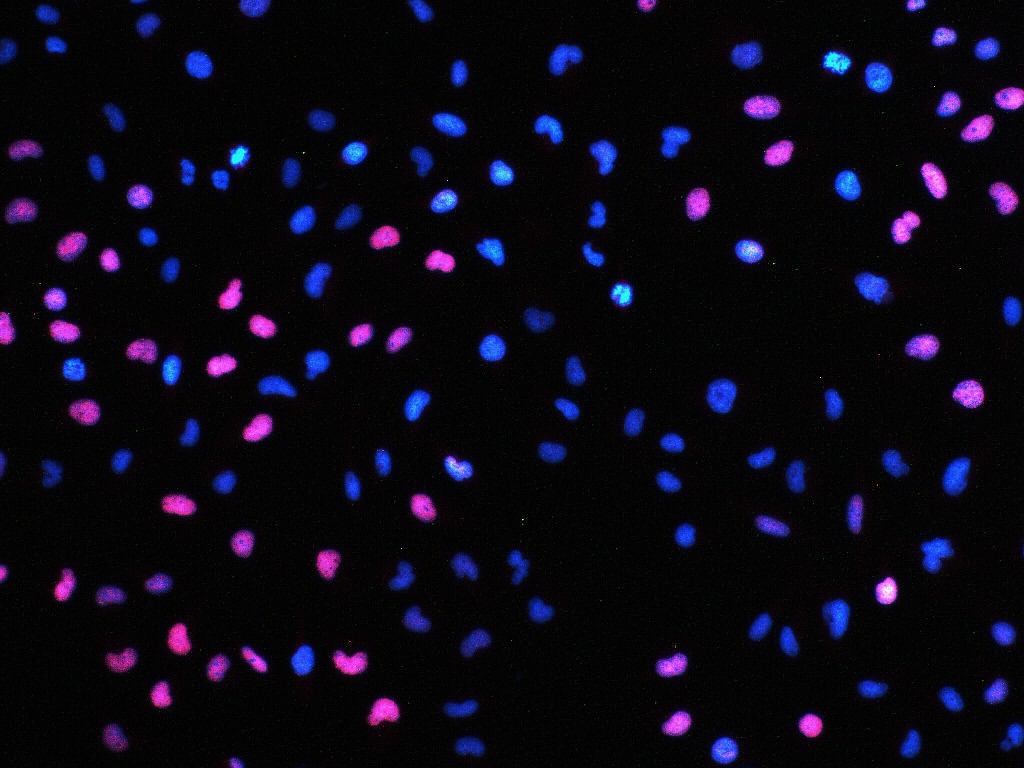

Supplement: Supplementary file 1 [file Data_Sheet_1.ZIP › 975640_SupMaterial/975640_SupMaterial/raw data frontiers/Figure 5/Figure 5A figures/Ox-LDL 200-1+2.JPG]

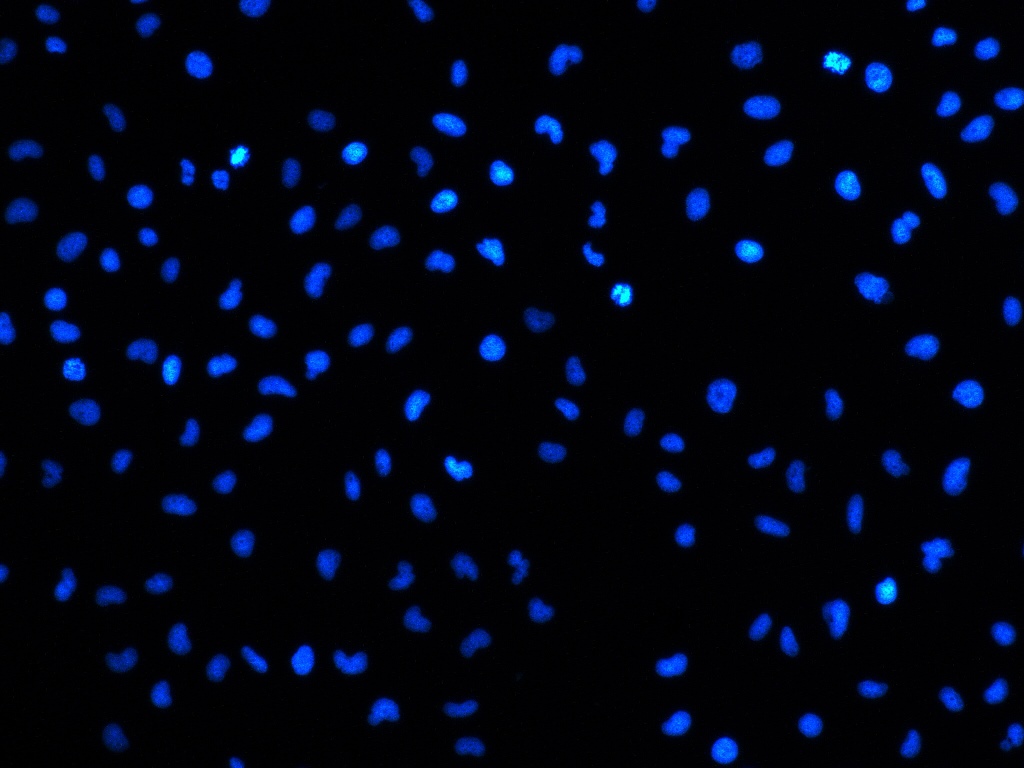

Supplement: Supplementary file 1 [file Data_Sheet_1.ZIP › 975640_SupMaterial/975640_SupMaterial/raw data frontiers/Figure 5/Figure 5A figures/Ox-LDL 200-1.jpg]

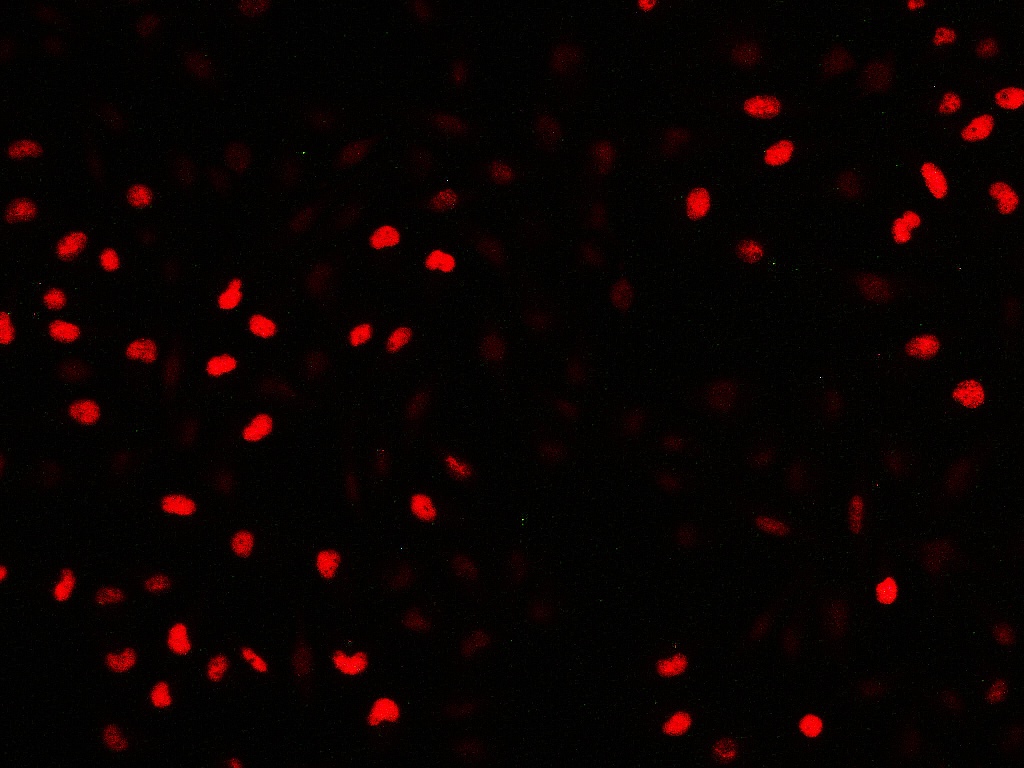

Supplement: Supplementary file 1 [file Data_Sheet_1.ZIP › 975640_SupMaterial/975640_SupMaterial/raw data frontiers/Figure 5/Figure 5A figures/Ox-LDL 200-2.jpg]

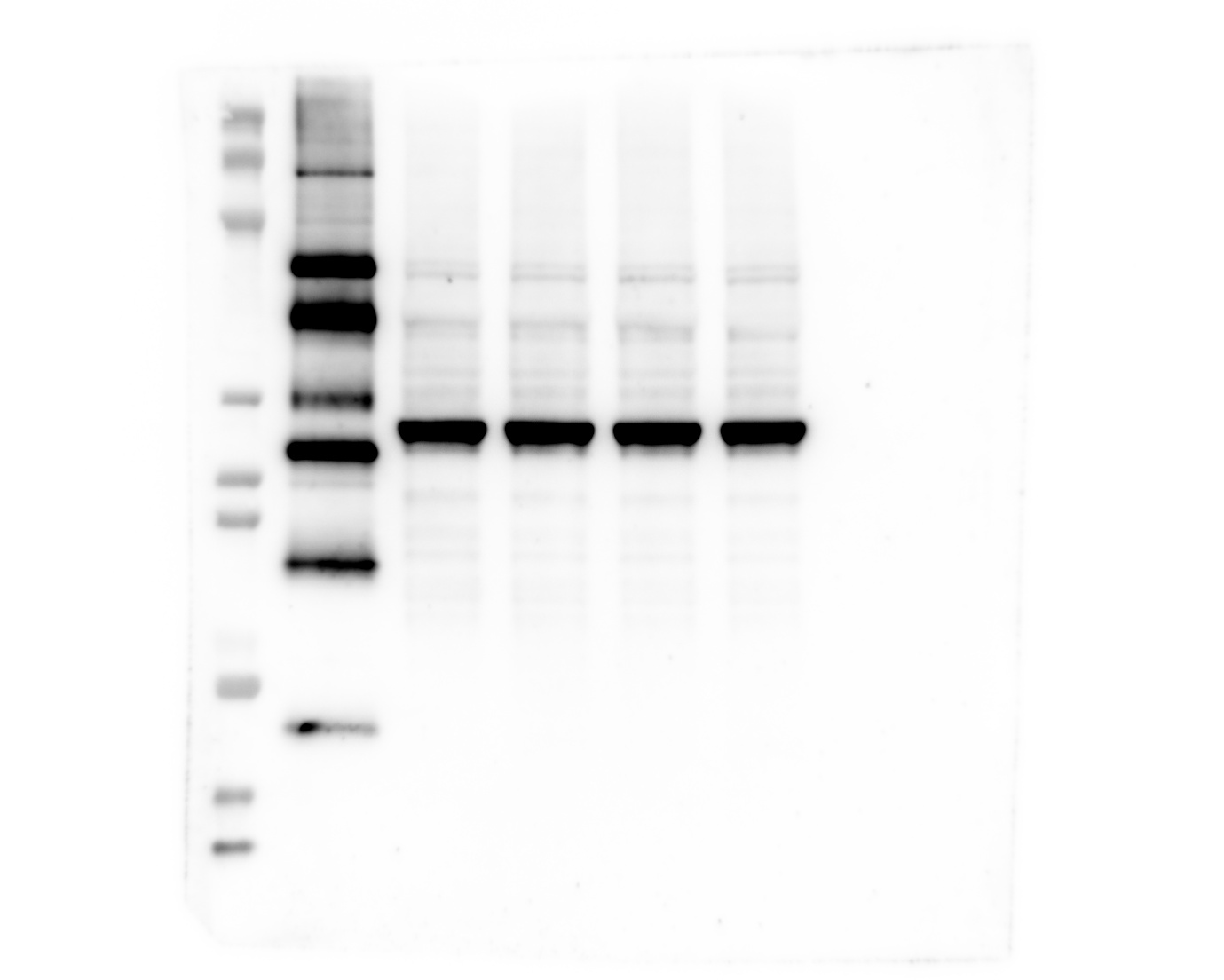

Supplement: Supplementary file 1 [file Data_Sheet_1.ZIP › 975640_SupMaterial/975640_SupMaterial/raw data frontiers/Figure 6/b-actin.jpg]

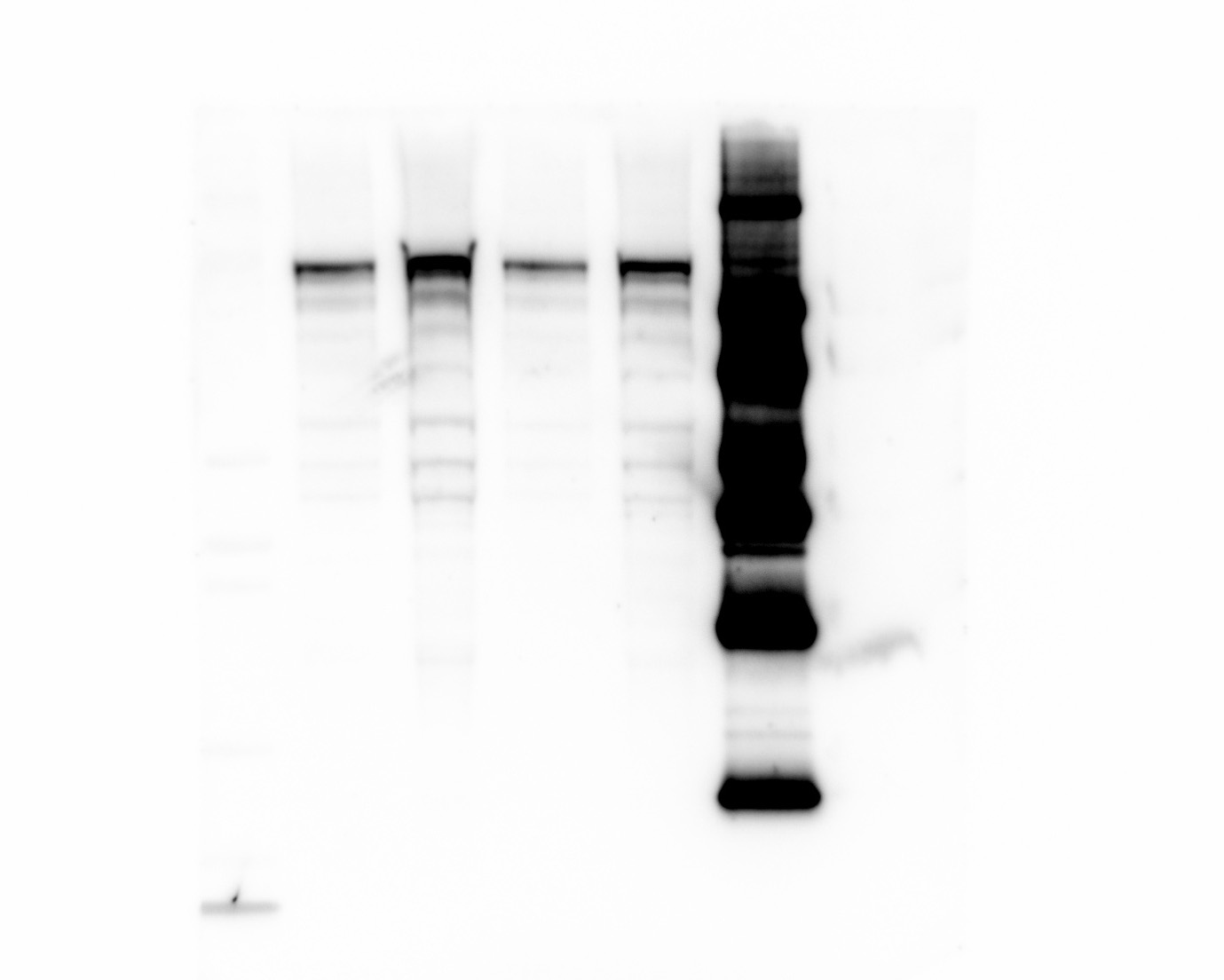

Supplement: Supplementary file 1 [file Data_Sheet_1.ZIP › 975640_SupMaterial/975640_SupMaterial/raw data frontiers/Figure 6/b-catenin.jpg]

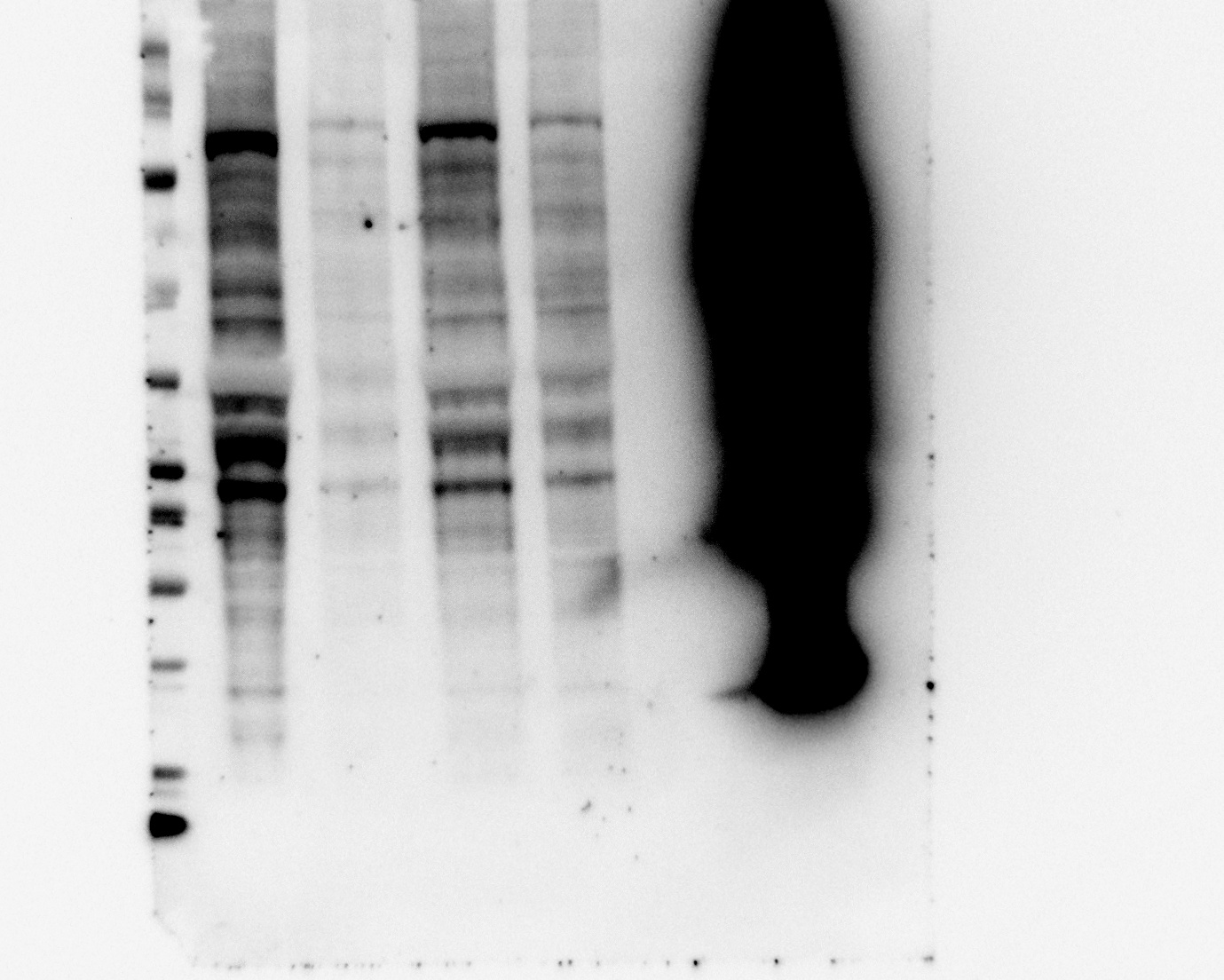

Supplement: Supplementary file 1 [file Data_Sheet_1.ZIP › 975640_SupMaterial/975640_SupMaterial/raw data frontiers/Figure 6/IGF1R.jpg]

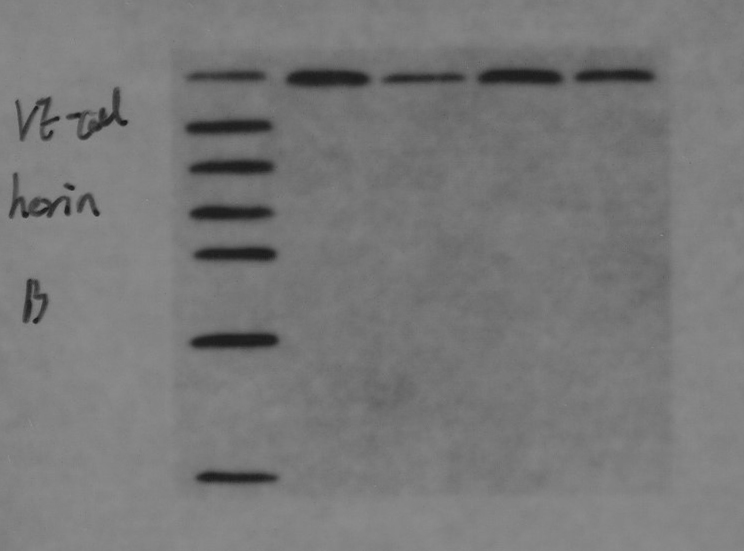

Supplement: Supplementary file 1 [file Data_Sheet_1.ZIP › 975640_SupMaterial/975640_SupMaterial/raw data frontiers/Figure 6/VE-cadherin-B.jpg]

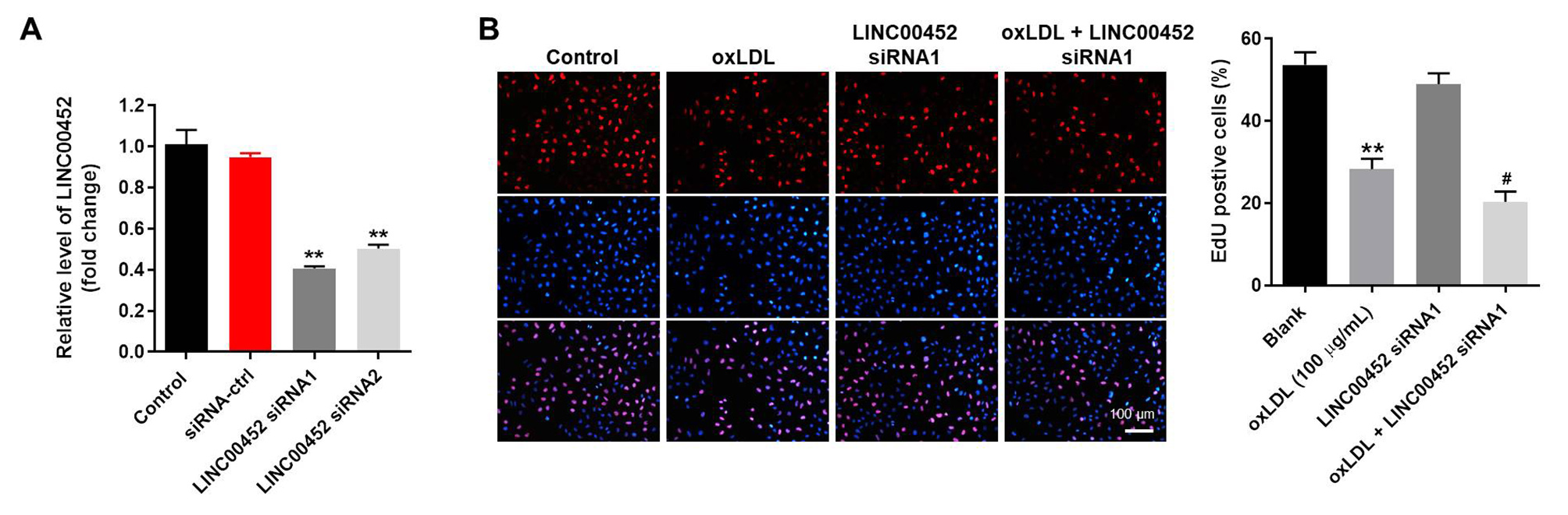

Supplement: Supplementary Figure 1 — LINC00452 knockdown further aggravated oxLDL-induced inhibition of HUVEC proliferation. (A) HUVECs were transfected with LINC00452 siRNA1 or siRNA2. The level of LINC00452 in HUVECs was detected by RT-qPCR. (B) HUVECs were treated with oxLDL, LINC00452 siRNA1 or oxLDL + LINC00452 siRNA2. The proliferation of HUVECs was tested by EdU staining. **P < 0.01 compared to control group. #P < 0.05 compared to oxLDL (100 μg/mL) group. [file Image_1.JPEG]
